# Novel 5-Nitrofuran-Tagged Imidazo-Fused Azines and Azoles Amenable by the Groebke–Blackburn–Bienaymé Multicomponent Reaction: Activity Profile against ESKAPE Pathogens and Mycobacteria

**DOI:** 10.3390/biomedicines10092203

**Published:** 2022-09-06

**Authors:** Alexander Sapegin, Elizaveta Rogacheva, Lyudmila Kraeva, Maxim Gureev, Marine Dogonadze, Tatiana Vinogradova, Petr Yablonsky, Saeed Balalaie, Sergey V. Baykov, Mikhail Krasavin

**Affiliations:** 1 Institute of Chemistry, Saint Petersburg State University, Saint Petersburg 199034, Russia; 2 Pasteur Institute of Epidemiology and Microbiology, 14 Mira Street, Saint Petersburg 197101, Russia; 3 Laboratory of Chemoinformatics and Bioinformatics, Sechenov First Moscow State Medical University, Moscow 119435, Russia; 4 Saint Petersburg Research Institute of Phthisiopulmonology, 2-4 Ligovsky Prospekt, Saint Petersburg 191036, Russia; 5 Peptide Chemistry Research Center, K. N. Toosi University of Technology, Tehran 19697, Iran; 6 Medical Biology Research Center, Kermanshah University of Medical Sciences Kermanshah, Kermanshah 67155, Iran; 7 School for Living Systems, Immanuel Kant Baltic Federal University, Kaliningrad 236041, Russia

**Keywords:** Groebke–Blackburn–Bienaymé multicomponent reaction, imidazo-fused azines and azoles, 5-nitrofurancarboxaldehyde, ESKAPE pathogens, antibacterial testing, antimycobacterial activity, flexible docking, strained ligand-protein interactions

## Abstract

A chemically diverse set of 13 5-nitrofuran-tagged heterocyclic compounds has been prepared via the Groebke–Blackburn–Bienaymé multicomponent reaction. The testing of these compounds against the so-called ESKAPE panel of pathogens identified an apparent lead compound—*N*-cyclohexyl-2-(5-nitrofuran-2-yl)imidazo[1,2-*a*]pyridine-3-amine (**4a**)—which showed an excellent profile against *Enterobacter cloacae, Staphylococcus aureus, Klebsiella pneumoniae,* and *Enterococcus faecalis* (MIC 0.25, 0.06, 0.25 and 0.25 µg/mL, respectively). Its antibacterial profile and practically convenient synthesis warrant further pre-clinical development. Certain structure-activity relationships were established in the course of this study which were rationalized by the flexible docking experiments in silico. The assessment of antitubercular potential of the compounds synthesized against drug sensitive H37v strain of *Mycobacterium tuberculosis* revealed little potential of the imidazo-fused products of the Groebke–Blackburn–Bienaymé multicomponent reaction as chemotherapeutic agents against this pathogen.

## 1. Introduction

Modern drug discovery entails the screening of chemically diverse sets of small-molecule compounds in order to discover suitable leads for subsequent medicinal chemistry optimization into drug candidates [1]. This approach requires that a large portion of druglike chemical space be sampled by the screening set in question and annotated against relevant target-based [2] or phenotypical [3] assays. The need for accessing as large a chemical diversity as possible within the confines of tangible, reasonably sized screening collections mandated that the library synthesis is planned and conducted in such a way that each library member is representative of a unique portion of the chemical space both at the scaffold and the periphery appendage level [4]. This goal is essentially the ideological core of the diversity-oriented synthesis [5] where highly efficient synthetic strategies based on atom-economical and multicomponent processes play the central role [6].

**Figure 1:**
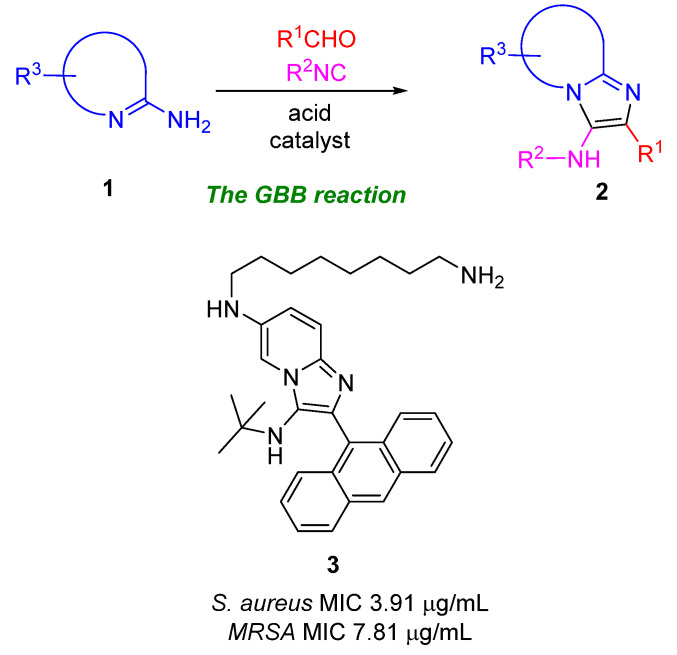
The Groeble–Blackburn–Bienaymé (GBB) reaction and relevant GBB reaction-derived antibacterial lead compound **3**.

In 1998, a new isocyanide-based multicomponent reaction was described by three independent research groups (Blackburn [7], Bienaymé [8], and Groebke [9]) which has been dubbed the Groeble–Blackburn–Bienaymé (GBB) reaction [10]. This reaction involves the reaction of amidine-like 2-aminoazines and azoles **1** with aldehydes and isocyanides to render *N*-bridgehead heterobicyclic compounds **2** in a one-pot format. Undoubtedly, it does allow to control both the scaffold and the periphery group diversity within a single chemical event. Considering the undisputed biological relevance of the imidazo-fused scaffolds **2** and the practical simplicity of the reaction directly catering to the needs of the diversity-oriented synthesis, the Groeble–Blackburn–Bienaymé reaction quickly became one of the gems in the toolbox for modern medicinally-spirited compound library development [11]. The productivity of the reaction in delivering valuable leads for drug discovery is illustrated by numerous success stories in such therapeutic and target areas as kinase inhibition [12], *M. tuberculosis* glutamine synthetase inhibition [13], antimicrobial and antifungal drug discovery [14], anticancer agents [15], dual soluble epoxide hydrolase (sEH)/ 5-lipoxygenase (5-LOX) inhibition [16], and, particularly relevant to the field of the present study, antibacterial lead generation resulting in the discovery lead compound **3** which showed efficacy against drug susceptible and methicillin-resistant *Staphylococcus aureus* [17] (Figure 1).

Although numerous various nitro heterocyclic moieties have manifested themselves as a source of antibacterial leads [18], the conjugation of classical nitrofurans to various heterocyclic motifs not only can render these compounds generally non-cytotoxic [19] but also can direct their activity towards a specific type of antibacterial property, that does not affect the microbiome at large [20]. Moreover, nitrofurans proved to be versatile synthetic intermediates en route to other furan derivatives that were accessed via nucleophilic substitution of the nitro group [21]. A few years ago, we [22,23] and others [24] have already explored this strategy successfully by conjugating privileged [25] aminoalkylimidazoles, 1,2,4-oxadiazoles, and pyrimidines to 5-nitrofuranoyl moiety, which resulted in compounds that were found to be antibacterial. Considering the intrinsic association of the GBB reaction-derived imidazo-fused scaffold with antibacterial activity, as demonstrated by the biological profile of compound **3**, combining the cluster of imidazo-fused scaffolds amenable by the GBB reactions with the known antibacterial pharmacophores such as 5-nitrofuryl moiety, surprisingly, has not been exploited to-date and clearly presents itself as a viable and well substantiated research opportunity. Herein, we report on the successful realization of this antibacterial drug design idea.

## 2. Materials and Methods

### 2.1. Organic Synthesis

#### 2.1.1. General Considerations

All commercial reagents and solvents were used without further purification, unless otherwise noted. THF for the synthesis was distilled over Na and stored under nitrogen over freshly activated molecular sieves 4 Å. The NMR spectra were recorded on a Bruker Avance III 400 spectrometer (^1^H: 400.13 MHz; ^13^C: 100.61 MHz; chemical shifts are reported as parts per million (δ, ppm)) (Bruker, Billerica, MA, USA); the residual solvent peaks were used as internal standards: 7.28 and 2.50 ppm for ^1^H in CDCl_3_ and DMSO-*d*_6_, respectively, 40.01 and 77.02 ppm for ^13^C in DMSO-*d*_6_ and CDCl_3_, respectively. Mass spectra were recorded on a Bruker Maxis HRMS-ESI-qT spectrometer (ESI ionization) (Bruker, Billerica, MA, USA). The melting points were determined in open capillary tubes on a Stuart SMP30 Melting Point Apparatus (Staffordshire, UK). Analytical thin-layer chromatography was carried out on Silufol UV-254 silica gel plates (Chemapol, Czech Republic) using appropriate mixtures of ethyl acetate and hexane. The compounds were visualized with short-wavelength UV light. Column chromatography was performed on silica gel 60 (230–400 mesh).

#### 2.1.2. General Procedure for the Synthesis of GBB Reaction Products **4a**–**m**

To the solution of the 2-aminoheteroarene **1a–m** (0.50 mmol) and 5-nitrofuran-2-carbaldehyde (71 mg, 0.6 mmol) in methanol (1 mL) was added isocyanide (0.6 mmol) and *p*-toluenesulfonic acid (17 mg, 0.1 mmol). After stirring at room temperature for 24 h, the solvent was removed by rotary evaporation under vacuum. The residue was taken up in water (10 mL) and extracted with ethyl acetate (10 mL). The organic phase was washed with water (2 × 10 mL), dried over anhydrous Na_2_SO_4_, filtered, and concentrated by rotary evaporation under vacuum. Column chromatography on SiO_2_ using 0→5% EtOAc in CH_2_Cl_2_ as eluent afforded the title compound.

##### N-Cyclohexyl-2-(5-nitrofuran-2-yl)imidazo[1,2-a]pyridin-3-amine (**4a**)

Yield 73%; brown solid; mp 191–193 °C; ^1^H NMR (400 MHz, DMSO-*d*_6_): *δ* 8.32 (d, *J* = 6.9 Hz, 1H), 7.85 (d, *J* = 3.9 Hz, 1H), 7.50 (d, *J* = 9.1 Hz, 1H), 7.29–7.22 (m, 1H), 7.17 (d, *J* = 3.9 Hz, 1H), 6.95 (t, *J* = 6.7 Hz, 1H), 5.04 (d, *J* = 7.4 Hz, 1H), 3.08 (tdt, *J* = 11.0, 7.6, 3.8 Hz, 1H), 1.94–1.77 (m, 2H), 1.78–1.61 (m, 2H), 1.59–1.46 (m, 1H), 1.44–1.28 (m, 2H), 1.26–1.08 (m, 3H) ppm; ^13^C{^1^H} NMR (101 MHz, CDCl_3_): *δ* 153.8, 151.2, 141.7, 130.5, 125.8, 124.4, 124.1, 117.7, 116.3, 112.8, 110.2, 57.2, 34.2 (2C), 25.8, 25.0 (2C) ppm; HRMS (ESI), *m*/*z* calcd for C_17_H_18_N_4_O_3_ [M+H]^+^ 327.1452, found 327.1456.

##### 6-Chloro-N-cyclohexyl-2-(5-nitrofuran-2-yl)imidazo[1,2-a]pyridin-3-amine (**4b**)

Yield 81%; dark orange solid; mp 247–250 °C; ^1^H NMR (400 MHz, DMSO-*d*_6_): *δ* 8.54 (d, *J* = 2.0 Hz, 1H), 7.86 (d, *J* = 4.0 Hz, 1H), 7.56 (d, *J* = 9.6 Hz, 1H), 7.29 (dd, *J* = 9.5, 2.0 Hz, 1H), 7.16 (d, *J* = 4.0 Hz, 1H), 5.16 (d, *J* = 7.8 Hz, 1H), 3.21–3.01 (m, 1H), 1.92–1.79 (m, 3H), 1.79–1.67 (m, 2H), 1.60–1.48 (m, 1H), 1.46–1.27 (m, 2H), 1.27–1.09 (m, 4H) ppm; ^13^C{^1^H} NMR (101 MHz, CDCl_3_): *δ* 153.1, 139.9, 131.1, 126.5, 124.8 (2C), 121.8, 120.1, 118.7, 116.2, 110.5, 57.2, 34.1 (2C), 25.7, 25.1 (2C) ppm; HRMS (ESI), *m*/*z* calcd for C_17_H_17_ClN_4_O_3_ [M+H]^+^ 361.1062, found 361.1065.

##### N-Cyclohexyl-2-(5-nitrofuran-2-yl)-6-(trifluoromethyl)imidazo[1,2-a]pyridin-3-amine (**4c**)

Yield 84%; orange solid; mp 240–243 °C; ^1^H NMR (400 MHz, DMSO-*d*_6_): *δ* 8.90 (d, *J* = 2.2 Hz, 1H), 7.86 (d, *J* = 3.9 Hz, 1H), 7.70 (d, *J* = 9.5 Hz, 1H), 7.45 (dd, *J* = 9.6, 1.8 Hz, 1H), 7.17 (d, *J* = 4.0 Hz, 1H), 5.42 (d, *J* = 8.0 Hz, 1H), 3.26–3.14 (m, 1H), 1.92–1.80 (m, 2H), 1.76–1.62 (m, 2H), 1.59–1.50 (m, 1H), 1.42–1.28 (m, 2H), 1.27–1.10 (m, 3H) ppm; ^13^C{^1^H} NMR (101 MHz, CDCl_3_): *δ* 152.8, 151.5, 140.9, 132.1, 129.7 (q, *J* = 262.5 Hz), 124.6 (q, *J* = 6.0 Hz), 123.7, 120.7 (q, *J* = 2.5 Hz), 118.9 (2C), 116.24 (d, *J* = 34.1 Hz), 110.9, 57.5, 34.1 (2C), 25.7, 25.1 (2C) ppm; HRMS (ESI), *m*/*z* calcd for C_18_H_17_F_3_N_4_O_3_ [M+H]^+^ 395.1326, found 395.1329.

##### N-Cyclohexyl-7-methyl-2-(5-nitrofuran-2-yl)imidazo[1,2-a]pyridin-3-amine (**4d**)

Yield 77%; dark brown solid; mp 203–206 °C; ^1^H NMR (400 MHz, CDCl_3_): *δ* 7.96 (d, *J* = 7.1 Hz, 1H), 7.48 (d, *J* = 3.9 Hz, 1H), 7.26 (d, *J* = 1.8 Hz, 1H), 7.03 (d, *J* = 3.8 Hz, 1H), 6.68 (dd, *J* = 7.1, 1.6 Hz, 1H), 3.86 (d, *J* = 7.7 Hz, 1H), 3.12–2.98 (m, 1H), 2.42 (s, 3H), 2.01–1.92 (m, 2H), 1.83–1.75 (m, 2H), 1.46–1.34 (m, 2H), 1.31–1.19 (m, 3H) ppm; ^13^C{^1^H} NMR (101 MHz, CDCl_3_): *δ* 154.1, 143.0, 136.4, 129.1, 124.3, 122.4, 115.9, 115.2, 114.4, 108.4, 77.2, 57.5, 34.1 (2C), 25.5, 25.0 (2C), 21.4 ppm; HRMS (ESI), *m*/*z* calcd for C_18_H_20_N_4_O_3_ [M+H]^+^ 341.1608, found 341.1605.

##### N-(tert-Butyl)-5-methyl-2-(5-nitrofuran-2-yl)imidazo[1,2-a]pyridin-3-amine (**4e**)

Yield 80%; reddish solid; mp 164–167 °C; ^1^H NMR (400 MHz, DMSO-*d*_6_): *δ* 7.85 (d, *J* = 4.0 Hz, 1H), 7.42–7.32 (m, 2H), 7.19 (dd, *J* = 9.0, 6.7 Hz, 1H), 6.67 (dt, *J* = 6.8, 1.2 Hz, 1H), 4.50 (s, 1H), 2.93 (s, 3H), 0.96 (s, 9H) ppm; ^13^C{^1^H} NMR (101 MHz, DMSO-*d*_6_): *δ* 153.8, 151.2, 143.8, 137.9, 130.3, 129.7, 126.3, 116.0, 115.7, 115.0, 112.2, 56.4, 29.5, 20.3 (3C) ppm; HRMS (ESI), *m*/*z* calcd for C_16_H_18_N_4_O_3_ [M+H]^+^ 315.1452, found 315.1457.

##### 8-(Benzyloxy)-N-(tert-butyl)-2-(5-nitrofuran-2-yl)imidazo[1,2-a]pyridin-3-amine (**4f**)

Yield 69%; reddish solid; mp 157–160 °C; ^1^H NMR (400 MHz, CDCl_3_): *δ* 7.91 (dd, *J* = 6.9, 0.9 Hz, 1H), 7.53–7.47 (m, 2H), 7.44 (d, *J* = 3.9 Hz, 1H), 7.41–7.29 (m, 4H), 7.24 (d, *J* = 4.0 Hz, 1H), 6.64 (t, *J* = 7.2 Hz, 1H), 6.47 (d, *J* = 7.5 Hz, 1H), 5.36 (s, 2H), 3.79–3.60 (m, 1H), 1.21 (s, 9H) ppm; ^13^C{^1^H} NMR (101 MHz, CDCl_3_): *δ* 146.1, 142.8, 132.7, 131.2, 123.9 (2C), 123.6, 123.4, 122.5 (2C), 112.5 (2C), 109.4, 107.3, 105.4, 98.9, 66.2, 52.4, 25.2 (3C). ppm; HRMS (ESI), *m*/*z* calcd for C_22_H_22_N_4_O_4_ [M+H]^+^ 407.1714, found 407.1717.

##### N-Cyclohexyl-2-(5-nitrofuran-2-yl)imidazo[1,2-a]pyrimidin-3-amine (**4g**)

Yield 63%; dark brown solid; mp 230–233 °C; ^1^H NMR (400 MHz, CDCl_3_): *δ* 8.56 (dd, *J* = 4.0, 2.0 Hz, 1H), 8.39 (dd, *J* = 6.9, 2.1 Hz, 1H), 7.49 (d, *J* = 3.8 Hz, 1H), 7.19 (d, *J* = 3.9 Hz, 1H), 6.91 (dd, *J* = 6.9, 4.1 Hz, 1H), 3.92 (d, *J* = 7.5 Hz, 1H), 3.15–2.99 (m, 1H), 2.03–1.91 (m, 2H), 1.86–1.71 (m, 2H), 1.51–1.35 (m, 2H), 1.35–1.21 (m, 4H) ppm; ^13^C{^1^H} NMR (101 MHz, CDCl_3_): *δ* 153.1, 150.8, 145.0, 130.8, 127.4 (2C), 125.8, 114.1, 110.2, 108.7, 57.85 34.1 (2C), 25.4 (2C), 24.8 ppm; HRMS (ESI), *m*/*z* calcd for C_16_H_17_N_5_O_3_ [M+H]^+^ 328.1404, found 328.1406.

##### N-Cyclohexyl-5,7-dimethyl-2-(5-nitrofuran-2-yl)imidazo[1,2-a]pyrimidin-3-amine (**4h**)

Yield 72%; dark brown solid; mp > 250 °C; ^1^H NMR (400 MHz, CDCl_3_): *δ* 7.46 (d, *J* = 3.9 Hz, 1H), 7.14 (d, *J* = 3.9 Hz, 1H), 6.45 (s, 1H), 3.62 (d, *J* = 7.8 Hz, 1H), 2.95 (s, 3H), 2.93–2.82 (m, 1H), 2.53 (s, 3H), 1.92 (d, *J* = 12.3 Hz, 2H), 1.75 (q, *J* = 4.3 Hz, 2H), 1.42–1.07 (m, 6H) ppm; ^13^C{^1^H} NMR (101 MHz, CDCl_3_): *δ* 161.2, 153.6, 151.5, 146.5, 145.7, 128.6, 127.3, 114.2, 111.3, 110.2, 61.0, 33.2 (2C), 25.6, 25.3 (2C), 24.55, 18.6 ppm; HRMS (ESI), *m*/*z* calcd for C_18_H_21_N_5_O_3_ [M+H]^+^ 356.1717, found 356.1719.

##### N-Cyclohexyl-6-(5-nitrofuran-2-yl)imidazo[2,1-b]thiazol-5-amine (**4i**)

Yield 51%; dark brown solid; mp 117–120 °C; ^1^H NMR (400 MHz, CDCl_3_): *δ* 7.47 (d, *J* = 3.9 Hz, 1H), 7.37 (d, *J* = 4.6 Hz, 1H), 6.86 (d, *J* = 4.6 Hz, 1H), 6.83 (d, *J* = 3.9 Hz, 1H), 4.26–4.17 (m, 1H), 3.21–3.09 (m, 1H), 2.05–1.95 (m, 2H), 1.88–1.75 (m, 2H), 1.43–1.23 (m, 6H) ppm; ^13^C{^1^H} NMR (101 MHz, CDCl_3_): *δ* 154.32, 146.2, 142.0, 136.4, 132.5, 116.98, 114.95, 113.58, 106.85, 57.2, 33.99 (2C), 25.50 (2C) ppm; HRMS (ESI), *m*/*z* calcd for C_15_H_16_N_4_O_3_S [M+H]^+^ 333.1016, found 333.1014.

##### N-(tert-Butyl)-2,3-dimethyl-6-(5-nitrofuran-2-yl)imidazo[2,1-b]thiazol-5-amine (**4j**)

Yield 53%; dark orange solid; mp 132–235 °C; ^1^H NMR (400 MHz, CDCl_3_): *δ* 7.44 (d, *J* = 3.9 Hz, 1H), 6.87 (d, *J* = 3.9 Hz, 1H), 3.38 (s, 1H), 2.60 (d, *J* = 1.2 Hz, 3H), 2.31 (q, *J* = 1.1 Hz, 3H), 1.15 (s, 9H) ppm; ^13^C{^1^H} NMR (101 MHz, CDCl_3_): *δ* 154.1, 146.6, 145.9, 130.7, 129.1, 125.1, 120.0, 114.5, 108.2, 56.3, 29.5 (3C), 12.7, 11.5 ppm; HRMS (ESI), *m*/*z* calcd for C_15_H_18_N_4_O_3_S [M+H]^+^ 335.1172, found 335.1176.

##### Ethyl 5-(cyclohexylamino)-3-methyl-6-(5-nitrofuran-2-yl)imidazo[2,1-b]thiazole-2-carboxylate (**4k**)

Yield 60%; dark orange solid; mp 188–191 °C; ^1^H NMR (400 MHz, CDCl_3_): *δ* 7.45 (d, *J* = 3.9 Hz, 1H), 6.90 (d, *J* = 3.8 Hz, 1H), 4.45–4.32 (m, 2H), 3.60 (d, *J* = 8.6 Hz, 1H), 3.05 (s, 3H), 2.98–2.85 (m, 1H), 2.05–1.94 (m, 2H), 1.88–1.73 (m, 2H), 1.49–1.15 (m, 8H) ppm; ^13^C{^1^H} NMR (101 MHz, CDCl_3_): *δ* 161.8, 153.0, 146.6, 138.5, 132.9, 128.2, 118.2, 115.3, 114.3, 108.4, 61.8, 60.5, 33.4 (2C), 25.4 (3C), 14.2, 12.4 ppm; HRMS (ESI), *m*/*z* calcd for C_19_H_22_N_4_O_5_S [M+H]^+^ 419.1384, found 419.1387.

##### Ethyl 2-(5-(tert-butylamino)-6-(5-nitrofuran-2-yl)imidazo[2,1-b]thiazol-3-yl)acetate (**4l**)

Yield 58%; dark oil; ^1^H NMR (400 MHz, CDCl_3_): *δ* 7.44 (d, *J* = 3.9 Hz, 1H), 6.91 (d, *J* = 3.9 Hz, 1H), 6.70 (s, 1H), 4.19–4.10 (m, 3H), 3.32 (s, 1H), 1.31 (t, *J* = 7.0 Hz, 3H), 1.14 (s, 9H) ppm; ^13^C{^1^H} NMR (101 MHz, CDCl_3_): *δ* 154.1, 146.6, 130.7, 129.1, 125.1, 120.0, 116.4, 114.5, 112.8, 108.2, 56.37 (2C), 29.5 (3C), 12.7, 11.5 ppm; HRMS (ESI), *m*/*z* calcd for C_17_H_20_N_4_O_5_S [M+H]^+^ 393.1227, found 393.1230.

##### N-(tert-Butyl)-2-methyl-6-(5-nitrofuran-2-yl)imidazo[2,1-b][1,3,4]thiadiazol-5-amine (**4m**)

Yield 32%; brown solid; mp 136–138 °C; ^1^H NMR (400 MHz, CDCl_3_): *δ* 7.44 (d, *J* = 3.9 Hz, 1H), 6.85 (d, *J* = 3.9 Hz, 1H), 4.02 (s, 1H), 2.73 (s, 3H), 1.35 (s, 9H) ppm; ^13^C{^1^H} NMR (101 MHz, CDCl_3_): *δ* 160.03, 153.73, 150.43, 143.24, 131.10, 125.48, 114.51, 107.50, 56.56, 30.18 (3C), 18.11 ppm; HRMS (ESI), *m*/*z* calcd for C_13_H_15_N_5_O_5_S [M+H]^+^ 322.0968, found 322.0965.

### 2.2. ESKAPE Pathogen Susceptibility Testing

Testing was conducted against the following microorganisms: *Enterococcus faecalis* (ATCC 29812), *Staphylococcus aureus* (ATCC 25912), *Klebsiella pneumoniae* (ATCC 19882), *Acinetobacter baumannii* (948^®^, patient-derived strain from the Pasteur Institute own collection), *Pseudomonas aeruginosa* (ATCC 27853), and *Enterobacter cloacae* (ATCC 13047) for compounds **4a–m**, nitrofurans nitrofurantoin [26] and furazidine [27] as well as ciprofloxacin (employed as a positive control) using the Kirby–Bauer disk diffusion test [28] under the Standard Operating Procedure of The European Committee on Antimicrobial Susceptibility Testing (EUCAST) [29]. Paper disks bearing 5 mg of the nitrofuran compounds and ciprofloxacin were used. Solutions of compounds **4a–m** made up in DMSO (1 mg/10 mL) were prepared and diluted to a total volume of 1 mL with deionized water. Aliquots of the resulting solutions (5 µL each) were added to a Petri dish containing Muller–Hilton agar that was inoculated with a bacterial suspension (McFarland OD ¼ 0.5). After the compound solution has dried off, the Petri dish was incubated at 37 °C for 18 h. The bacterial growth inhibition zone diameter around the disc with ciprofloxacin or the compounds’ dried solution circular spot indicated the general susceptibility to a drug being assessed. Thereupon, minimum inhibitory concentrations (MIC, µg/mL) were determined using serial broth dilutions [30]. All measurements were done in triplicate.

### 2.3. Testing against Mycobacterium tuberculosis

The testing was performed as described previously [31]. All the measurements were done in triplicate.

### 2.4. Molecular Modeling

#### 2.4.1. Target Selection

As the potential targets for observed nitrofuran derivatives **4a–m** the following two proteins were selected: *Escherichia coli* nitroreductase NfsB (PDP 1YKI) and oxygen-insensitive NADPH nitroreductase NfsA (PDP 7NB9). These proteins have been investigated as molecular targets for nitrofuran-based drugs [32]. The main reference interactants in this case are nitrofurazone [33] or nitrofurantoin [26], respectively.

#### 2.4.2. Protein and Ligand Structure Preparation

All protein structures were downloaded from the RCSB protein data bank [34] and preprocessed with use of Schrodinger Protein prepwizard. Invalid bond orders, protonation, missing residues, or residue sidechains were corrected in this step [35]. The geometry of ligands was generated with the use of LigPrep module. All of the manipulations with molecules performed in OPLS4 forcefield [36]. For calculations Schrodinger Suite version 2021-4 was used.

#### 2.4.3. Molecular Docking

Ligand binding poses were calculated with the use of the induced-fit docking method [37]. Unlike molecular docking, this method implies the flexibility of the protein structure. The docking grid box was calculated in accordance with the reference ligand positioning and the ligand size (grid positioning on complexed ligand centroid, maximal grid side size was 12 Å). The flexibility of the amino acids was taken into account within the 5 Å radius from the ligand atoms (not centroids). The best-fitting pose was selected manually by evaluating the reproducibility of interactions of the nitrofuran moiety in the reference ligand that was present in the protein active site (as in PDB file).

#### 2.4.4. Molecular Mechanics with Generalized Born and Surface Area Solvation (MM-GBSA)

For each ligand’s binding pose, the Gibbs free energy (ΔG) was calculated in the presence of implicit solvent. The calculations were performed with the use of MM-GBSA (Molecular mechanics with generalized Born and surface area solvation) method [38]. The point of interest was the strain energy value distribution which indicated the most strained protein-ligand interactions. This parameter was seen as able to explain the absence of the correct interaction of the ligand with the protein and, consequently, the diminished biological activity.

## 3. Results

### 3.1. Compound Synthesis

**Figure 2:**
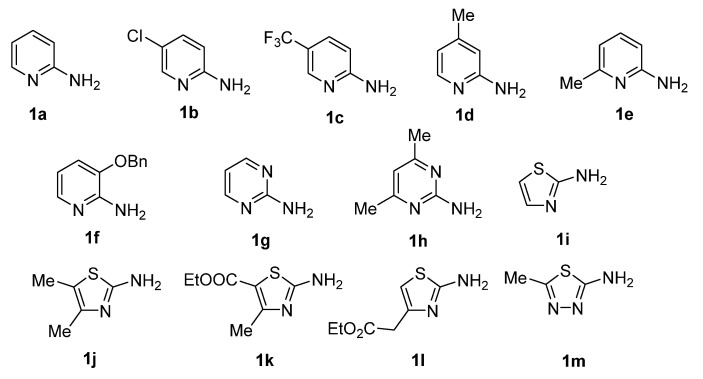
The diversity of 2-aminoazines and -azoles **1a–m** that were employed in the GBB synthesis of 5- nitrofuran-tagged compounds that were investigated in this work.

In order to explore the maximum chemical diversity within a limited compound set—and yet to be able to make pairwise comparison of the structure-activity relationships, we chose to place the 5-nitrofuran moiety within the aldehyde component, use only two fairly generic isocyanides (cyclohexyl and *tert*-butyl), and put a particular emphasis on the diversity of substituted 2-aminoazine and -azole inputs **1**, employing a new substrate out of 13 (**1a–m**) to create each of the GBB reaction products (Figure 2).

**Scheme 1:**
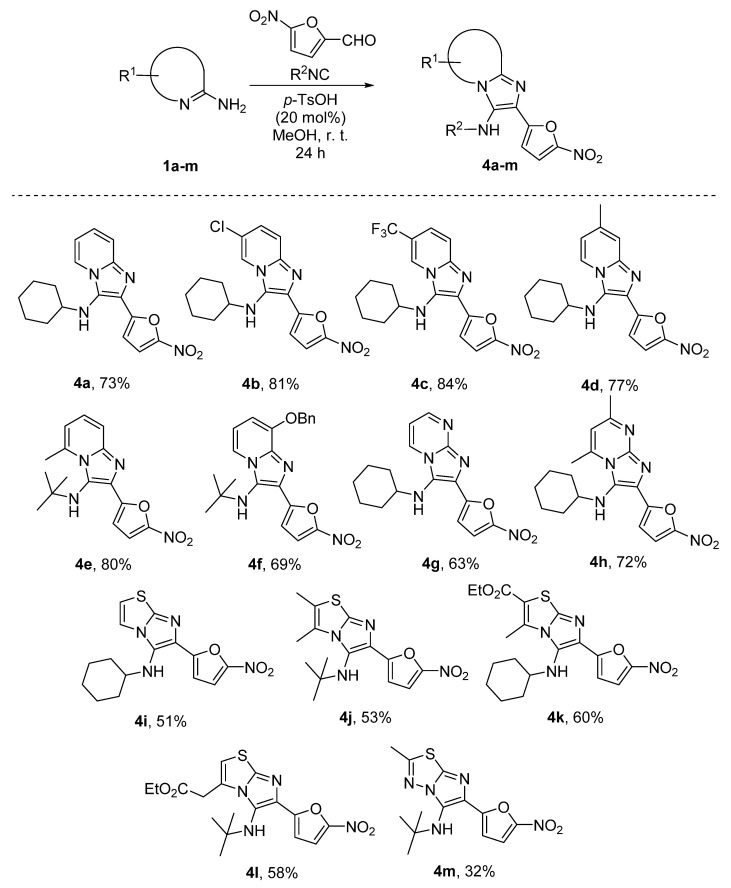
Synthesis of imidazo-fused products of the GBB reaction with **1a–m** showing the structure and percentage yield of each compound.

A unique substrate input (**1a–m**) was employed in the preparation of each of the GBB products (**4a–m**) by mixing equimolar amounts of **1**, 5-nitrofurancarboxaldehyde and an isocyanide (*c*-HexNC or *t*-BuNC) in methanol in the presence of 20 mol% of *p*-toluenesulfonic acid catalyst [39]. The reactions were complete within 24 h at room temperature. Following a brief aqueous workup, the target imidazo-fused GBB reaction products **4a–m** were isolated chromatographically in moderate to excellent yield (Scheme 1). The yields of the GBB reaction products that were derived from 2-aminoazoles were markedly lower compared to their 2-azine-derived counterparts.

### 3.2. Activity against ESKAPE Pathogens

Nitrofurans **4a–m** were tested against Gram-positive (*S. aureus* and *E. faecium*) or Gram-negative (*E. cloacae*, *P. aeruginosa*, *A. baumannii*, *K. pneumoniae*) pathogens belonging to the so-called ESKAPE panel [40] of highly virulent and antibiotic resistant bacterial pathogens which are, throughout the world, the major cause of life-threatening nosocomial or hospital-acquired infections in immunocompromised and critically ill patients who are most at risk [41]. There were two clinically used nitrofuran antibiotics—nitrofurantoin [26] and furazidine [27] as well as broad-spectrum fluoroquinoline antibiotic ciprofloxacin [42] that were used as positive controls and comparators. The compounds were initially screened at a single concentration to determine the presence and the diameter of the bacterial growth inhibition zone around the drug-treated disk. Those compounds that displayed growth inhibition were tested in serial dilution mode to determine the minimum inhibitory concentration (MIC) (Table 1).

### 3.3. Activity against Mycobacterium tuberculosis

All 13 compounds **4a–m** were tested against *Mycobacterium tuberculosis* H37Rv drug-sensitive strain relative to isoniazid that was employed as a positive control. As it follows from the minimum inhibitory concentration (MIC) data that are summarized in Table 2, most of the compounds were either inactive or only weakly active against this pathogen except for *N*-cyclohexyl-6-(5-nitrofuran-2-yl)imidazo[2,1-*b*]thiazol-5-amine (**4i**) which showed MIC value of 6.2 µg/mL.

### 3.4. Molecular Modeling

**Figure 3:**
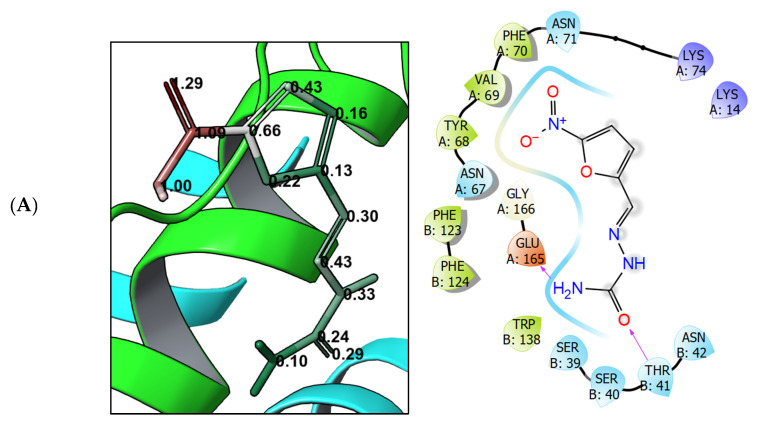

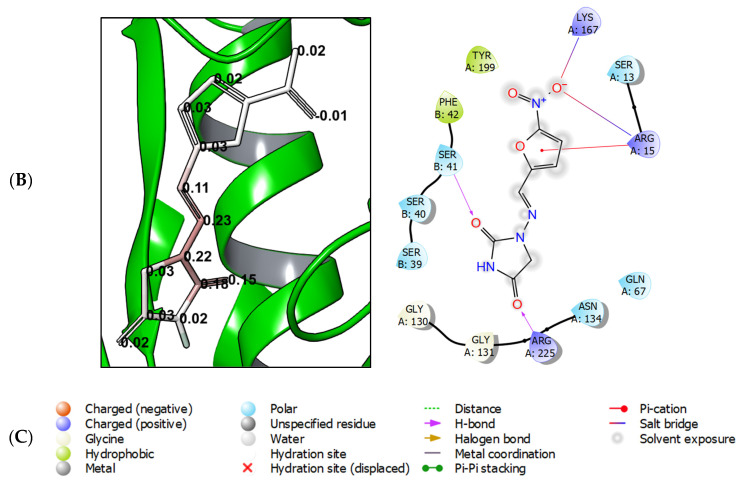
Strain energies and interaction diagrams of (**A**) nitrofurazone re-docked into the structure of *E. coli* nitroreductase NfsB (PDB 1YKI) and (**B**) nitrofurantoin re-docked into the structure of oxygen-insensitive NADPH nitroreductase NsfA (PDB 7NB9); (**C**) ligand interactions diagram legend.

The crystal structures of the two targets stipulated for nitrofurans—*E. coli* nitroreductase NfsB (PDB 1YKI) and oxygen-insensitive NADPH nitroreductase NfsA (PDB 7NB9) contain nitrofurazone and nitrofurantoin as bound ligands, respectively. In order to validate the docking protocol, these ligands were re-docked into the binding site. The docking poses that were thus obtained (allowing for determination of ligand strain components as well as key interactions with the target) reproduced the position of the ligand in the PDB structures with RMSD < 1.5 Å (Figure 3).

Having validated the docking protocol, we proceeded to perform docking of four selected GBB reaction-derived nitrofuran compounds (**4a–d**) two of which (**4b** and **4c**), surprisingly, showed no activity against the ESKAPE panel of pathogens while the other two (**4a** and **4d**) were quite active (except for no activity observed against *A. baumannii* and *P. aeruginosa* for all nitrofurans including the reference compounds). As all of the docked compounds showed good binding affinity according to the initially determined docking score values which did not allow differentiating between the active and inactive pair. Hence, we proceeded to switch to the induced-fit docking as described in the Materials and Methods section. With this approach, we observed significant differences between the active compounds (**4a** and **4d**) as well the nitrofuran reference ligands and inactive compounds (**4b** and **4c**) in their total calculated system energy as well as MM-GBSA Gibbs free energy (ΔG) values which were markedly higher for inactive protein-ligand complexes with both *E. coli* nitroreductase NfsB and oxygen-insensitive NADPH nitroreductase NfsA (Table 3).

**Figure 4:**
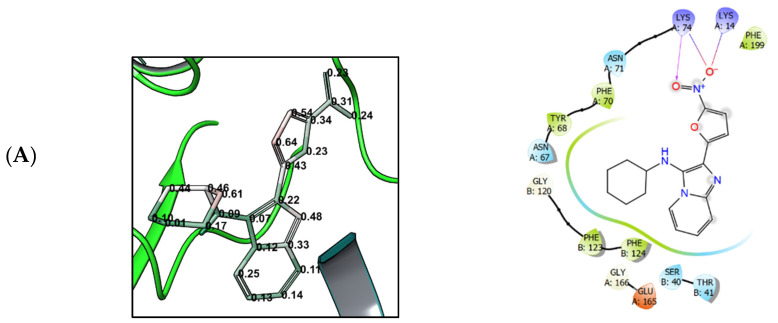

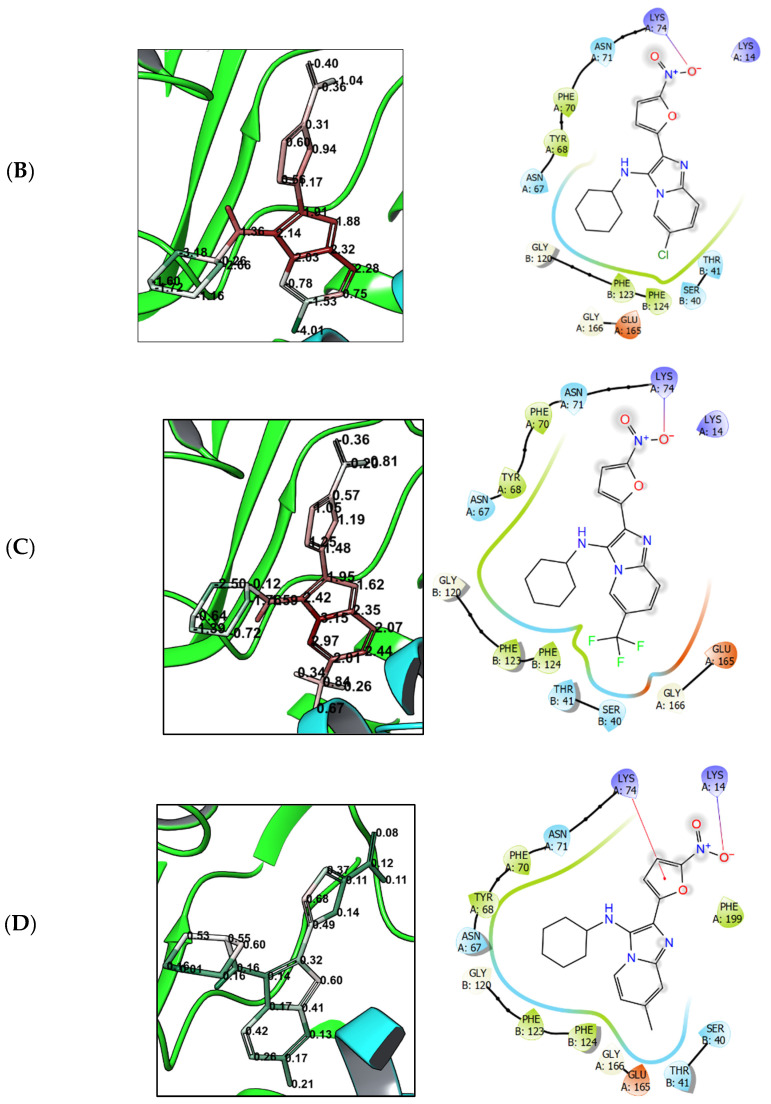
Strain energies and interaction diagrams of compounds **4a** (**A**), **4b** (**B**), **4c** (**C**), and **4d** (**D**) that were docked into the structure of *E. coli* nitroreductase NfsB (PDB 1YKI) using the flexible docking protocol.

**Figure 5:**
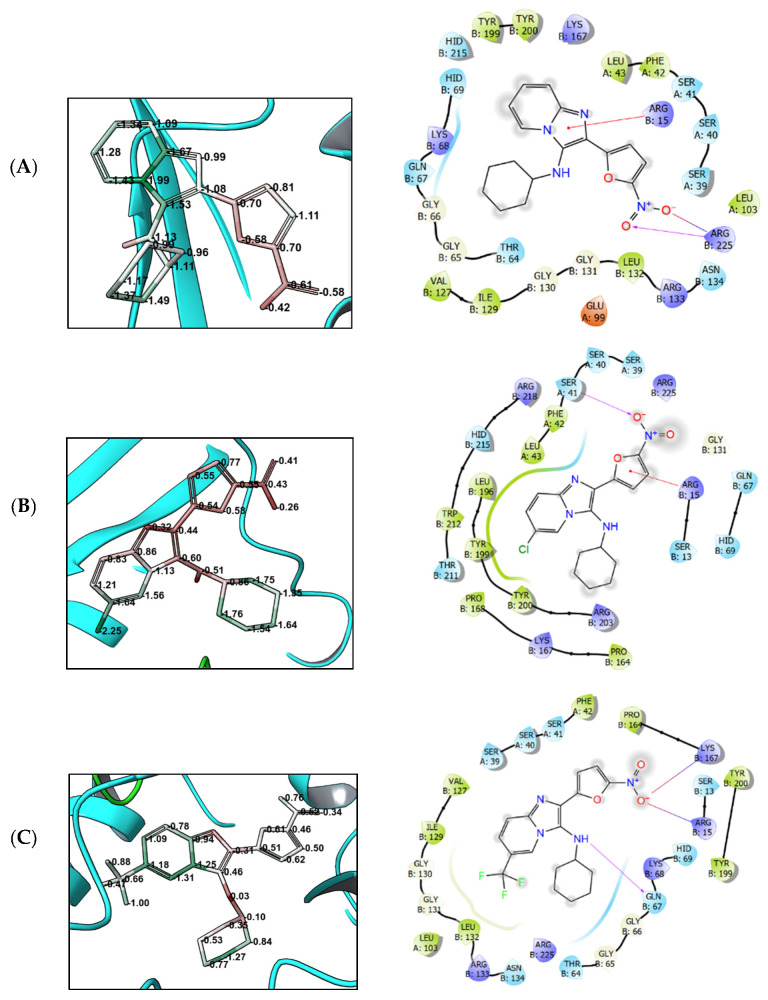

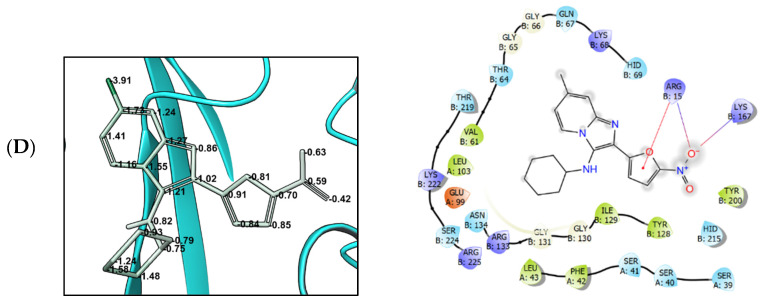
Strain energies and interaction diagrams of compounds **4a** (**A**), **4b** (**B**), **4c** (**C**), and **4d** (**D**) that were docked into the structure of oxygen-insensitive NADPH nitroreductase NsfA (PDB 7NB9) using the flexible docking protocol.

The binding poses of ligands **4a–d** in *E. coli* nitroreductase NfsB (PDB 1YKI) (Figure 4) and oxygen-insensitive NADPH nitroreductase NsfA (PDB 7NB9) (Figure 5) allowed analyzing the free energy distribution—in particular, the energies of strained ligand-protein contacts. This also allowed us to understand the components that contributed most to the differences between the active (**4a** and **4d**) and inactive (**4b** and **4c**) compounds.

In the case of *E. coli* nitroreductase NfsB, the addition of the chloro (**4b**) or the trifluoromethyl (**4c**) substituents induced the rearrangement of the ligand binding mode due to the increased hydrophobic interactions with Phe123 and Phe124. This also leads to a noticeable increase in the strain energy per atom of the imidazo[1,2-*a*]pyridine scaffold as well as the molecular periphery (Figure 4B,C increased strain indicated by the numbers as well as color change from mostly green in Figure 4A,D).

In the case of the oxygen-insensitive NADPH nitroreductase NsfA, the difference in the strain energy is less pronounced. However, compounds **4b** and **4c** still stand out in terms of the per-atom strain energy. The presence of the chloro of the trifluoromethyl substituents acts as a powerful hydrophobic anchor which stimulates the hydrophobic contacts with multiple hydrophobic residues in the enzyme’s binding pocket: aromatic amino acids such as Trp212, Phe42, Tyr199, and Tyr200 as well as non-polar Leu43 and Leu196. This exceedingly large number of lipophilic contacts induces the increase in the strain energy which eventually causes the ligand to migrate and bind in an unfavorable pose. This is likely what leads to the loss of the antibacterial activity due to the exclusion of the nitrofuran moiety from the enzyme’s catalytic center.

## 4. Discussion

The synthesis of compounds **4a–m** (see Supplementary Materials) is based on the GBB multicomponent reaction and commercially available starting materials. This makes any leads that surface from the present study particularly attractive from the development perspective as bringing up larger quantities of these compounds for in vivo efficacy and other preclinical profiling will not present a problem. Although currently nitrofurans such as furazidine, nitrofurantoin, and nitrofurazone are mostly used as topical medicines, their unfavorable activity profile and conjugation of nitrofuran moiety to druglike cores in the absence of apparent toxophores has been shown to deliver candidate molecules that are devoid of systemic toxicity [43]. This can potentially render the lead nitrofurans that were identified through this study as candidates for the development as orally acting anti-infectives. In this regard, compounds **4a–m** hold a great promise as they are entirely devoid of any violations of the rules for drug-likeness as defined by Lipinski [44] (Table 4).

As it can be seen from the antibacterial data that are summarized in Table 1, all of the compounds **4a–m** displayed a distinctly ‘nitrofuran-like’ profile in the sense that, similar to nitrofurantoin and furazidine, none of these compounds showed any activity towards *A. baumannii* and *P. aeruginosa*. The other apparent—and somewhat surprising—result that is shown in Table 1 is the total absence of antibacterial activity of compounds **4b** and **4c** across the entire ESKAPE panel. Besides these gashing voids in the activity profile—and a few other instances of inactivity (*cf.* **4c–d**, **4h–I**, and **4k** against *E. cloacae*; **4f** against *K. pneumoniae*; **4d–e** and **4l–m** against *E. faecalis*)—compounds **4**, in general, showed a superior activity profile relative to the two nitrofuran comparators and a comparable activity profile to that of broad-spectrum fluoroquinoline antibiotic ciprofloxacin. In terms of the overall activity profile against all four pathogens that wereaffected by nitrofurans—*E. cloacae* (G−), *S. aureus* (G+), *K. pneumonia* (G−), and *E. faecalis* (G+)—compound **4a** presented itself as a convincing lead displaying low MIC values against these bacteria. On top of the distinctly simple, one-step synthesis of this compound from readily available commercial precursors, this compound is apparently attractive from the further development perspective considering the low-cost and convenience of bringing up larger quantities of this candidate compound for subsequent in vivo efficacy and other preclinical profiling.

At the same time, it became clear that substitution of the imidazo[1,2-*a*]pyridine core with lipophilic groups such as chloro (**4b**) or trifluoromethyl (**4c**) negatively impacts on the antibacterial activity of the GBB reaction-derived nitrofurans. As demonstrated by the flexible docking experiments, these compounds differ from their active counterparts (**4a** and **4d**) in the increased per-atom ligand strain when bound to either *E. coli* nitroreductase NfsB or oxygen-insensitive NADPH nitroreductase NsfA. This interferes with the correct binding of the nitrofuran ligand in the enzymes’ active site and can even lead to a complete change of the binding mode which is the likely reason for the observed loss of the antibacterial activity.

Compounds **4a–m** showed little potential as representatives of an antitubercular chemotype with the majority of compounds having no or only a weak activity. One of these compounds (**4i**), however, displayed an MIC of 6.2 µg/mL. Considering that its thiadiazole congeners **4j–l** and thiadiazole **4m** all displayed much weaker activities, **4i** clearly stands alone as a potential antitubercular lead whose activity might be optimized further by the manipulation of the isocyanide-derived periphery.

## 5. Conclusions

We have reported synthesis, via the Groebke–Blackburn–Bienaymé multicomponent reaction, of a chemically diverse set of imidazo-fused azines and azoles containing a 5-nitrofuran moiety. These compounds were profiled for the antibacterial activity against Gram-positive (*S. aureus* and *E. faecalis*) and Gram-negative (*E. cloacae*, *P. aeruginosa*, *A. baumannii*, *K. pneumoniae*) pathogens belonging to the so-called ESKAPE panel, the highly virulent and antibiotic-resistant bacterial pathogens. As the other two nitrofuran comparator compounds (furazidine and nitrofurantoin), the newly synthesized 5-nitrofuran-tagged GBB reaction products did not show any activity towards *A. baumannii* and *P. aeruginosa*. However, towards the other four pathogens, the activity level significantly exceeded that of furazidine and nitrofurantoin and was en par with the antibacterial potency of ciprofloxacin, a broad-spectrum fluoroquinolone antibiotic. The only notable exception was presented by the two compounds containing lipophilic (chloro or trifluoromethyl) substituents in position 6 of the imidazo[1,2-*a*]pyridine core. These two compounds showed no activity whatsoever against any of the ESKAPE pathogens. As we hypothesized that the observed activity was mediated by nitroreductase enzymes, we performed flexible docking of these two inactive compounds as well as two closely related active ones into the active site of *E. coli* nitroreductase NfsB and oxygen-insensitive NADPH nitroreductase NsfA. The calculation of the total system energy for the ligands that were bound to the two enzymes showed a significant increase in the energy for the compounds which were inactive compared to the active ones as well as the enzymes’ native nitrofuran ligands. Detailed analysis of the ligand-protein interactions allowed us to conclude that this increase in energy is likely due to an increase in the ligand strain that is bound to the protein. This affects the correct binding of the nitrofuran moiety and can even cause the ligands to completely change its pose within the enzyme’s active site, which is the likely reason for the observed loss of activity. Altogether, a new antibacterial lead—*N*-cyclohexyl-2-(5-nitrofuran-2-yl)imidazo[1,2-*a*]pyridin-3-amine (compound **4a**) was identified which showed an excellent profile against *E. cloacae, S. aureus, K. pneumoniae,* and *E. faecalis* (MIC 0.25, 0.06, 0.25, and 0.25 µg/mL, respectively). This profile as well as its distinctly practical synthesis from commercially available precursors clearly warrants its further pre-clinical investigation.

The testing of the same set of compounds against the drug-sensitive H37v strain of *Mycobacterial tuberculosis* revealed the compounds’ low potential as antitubercular agents, compared to their outstanding activity against ESKAPE pathogens. Only one of the compounds (**4i**) displayed an MIC value of 6.2 µg/mL signifying itself as a starting point for further optimization.

## Figures and Tables

**Table 1:** Antibacterial activity (disk diffusion method inhibition zone (IZ, mm) and minimal inhibitory concentration (MIC, µg/mL) of compounds **4a–m**, nitrofurans furazidine and nitrofurantoin as well as ciprofloxacin (positive controls) against the ESKAPE panel of pathogens (E1 = *Enterobacter cloacae* (G−), S = *Staphylococcus aureus* (G+), K = *Klebsiella pneumoniae* (G−), A = *Acinetobacter baumannii* (G−), P = *Pseudomonas aeruginosa* (G−), E2 = *Enterococcus faecalis* (G+)); NT—not tested. The compounds that were not tested or having no activity are marked red. The MIC values are mean from three different assays (errors were in the range of ±5–10% of the reported values).

Compound	E1		S		K		A		P		E2	
	IZ	MIC	IZ	MIC	IZ	MIC	IZ	MIC	IZ	MIC	IZ	MIC
**4a**	17	0.25	17	0.06	7	0.25	0	NT	0	NT	9	0.25
**4b**	0	NT	0	NT	0	NT	0	NT	0	NT	0	NT
**4c**	0	NT	0	NT	0	NT	0	NT	0	NT	0	NT
**4d**	0	NT	10	0.43	6	1.75	0	NT	0	NT	0	NT
**4e**	14	0.45	15	0.23	7	0.45	0	NT	0	NT	0	NT
**4f**	11	0.78	11	1.55	0	NT	0	NT	0	NT	13	1.55
**4g**	10	1	17	0.5	8	0.5	0	NT	0	NT	13	0.5
**4h**	0	NT	9	0.43	7	0.43	0	NT	0	NT	0	NT
**4i**	0	NT	16	0.39	6	0.78	0	NT	0	NT	9	1.55
**4j**	12	2.1	19	0.53	6	2.1	0	NT	0	NT	13	1.05
**4k**	0	NT	18	0.66	9	0.83	0	NT	0	NT	15	0.66
**4l**	15	0.93	9	0.93	5	1.85	0	NT	0	NT	0	NT
**4m**	7	0.61	11	0.61	9	2.45	0	NT	0	NT	0	NT
Furazidine	13	32	23	8	14	32	0	NT	0	NT	21	2
Nitrofurantoin	15	8	21	4	12	64	0	NT	0	NT	24	4
Ciprofloxacin	NT	1.25	NT	1.25	NT	0.6	NT	2.5	NT	0.6	NT	0.3

**Table 2:** Activity of compounds **4a–m** against *Mycobacterium tuberculosis* H37Rv drug-sensitive strain. The most active compound (**4i**) is marked green.

Compound	MIC (µg/mL)	Compound	MIC (µg/mL)
**4a**	>100	**4h**	100
**4b**	>100	**4i**	6.2
**4c**	>100	**4j**	50
**4d**	>100	**4k**	50
**4e**	50	**4l**	100
**4f**	>100	**4m**	100
**4g**	100	Isoniazid	0.10

**Table 3:** Total system energy as well as MM-GBSA free Gibbs energy of compounds **4a–d** that were docked into *E. coli* nitroreductase NfsB (PDB 1YKI) and oxygen-insensitive NADPH nitroreductase NsfA (PDB 7NB9) using flexible docking protocol [native ligand = nitrofurazone (1YKI) or nitrofurantoin (7NB9)]. Comopounds with good binding energy parameters are marked green; compounds with poor binding energy parameters are marked orange.

Compound	Total System Energy (kcal/mol)		MM-GBSA ΔG (kcal/mol)	
	1YKI	7NB9	1YKI	7NB9
native-ligand	−17,665.90	−19,712.29	−75.40	−47.54
**4a**	−17,677.63	−19,737.57	−62.04	−51.57
**4b**	−15,897.41	−17,646.36	−11.97	−27.15
**4c**	−15,842.04	−17,638.79	−15.63	−34.85
**4d**	−17,641.98	−19,842.16	−58.44	−53.58

**Table 4:** Molecular parameters of compounds **4a–m** defining drug-likeness according to Lipinski [44] [MW—molecular weight (<500), cLogP—calculated lipophilicity characteristic defined as 1-octanol/water partitioning coefficient (<5.0), HBA—number of hydrogen bond acceptors (<10), HBD—number of hydrogen bond donors (<5), NRotB—number of rotatable bonds (<10)]; calculated using Molinspiration Cheminformatics tools [45].

Compound	MW	cLogP	HBA	HBD	NRotB
**4a**	326.4	4.08	7	1	4
**4b**	360.8	4.71	7	1	4
**4c**	394.4	4.93	7	1	5
**4d**	340.4	4.49	7	1	4
**4e**	314.4	3.20	7	1	4
**4f**	406.4	4.97	8	1	7
**4g**	327.3	3.16	8	1	4
**4h**	355.4	3.82	8	1	4
**4i**	332.4	3.82	7	1	4
**4j**	334.4	3.54	7	1	4
**4k**	392.4	3.77	9	1	7
**4l**	392.4	3.76	9	1	8
**4m**	321.4	2.48	8	1	4

## Data Availability

Not applicable.

## Supplementary Materials

The following supporting information can be downloaded at: https://www.mdpi.com/article/10.3390/biomedicines10092203/s1. Copies of ^1^H and ^13^C NMR spectra.
