# Supplementary material for: Novel 5-Nitrofuran-Tagged Imidazo-Fused Azines and Azoles Amenable by the Groebke–Blackburn–Bienaymé Multicomponent Reaction: Activity Profile against ESKAPE Pathogens and Mycobacteria
Source: Biomedicines. 2022 Sep 6;10(9):2203. doi: 10.3390/biomedicines10092203 (PMC9496245; doi:10.3390/biomedicines10092203)

Supporting Information for

Novel 5-Nitrofuran-Tagged Imidazo-Fused Azines and Azoles Amenable by the Groebke–Blackburn–Bienaymé Multicomponent Reaction: Activity Profile against ESKAPE Pathogens and Mycobacteria

Alexander Sapegin [1], Elizaveta Rogacheva [2], Lyudmila Kraeva [2], Maxim Gureev [3], Marine Dogonadze [4], Tatiana Vinogradova [4], Petr Yablonsky [4], Saeed Balalaie [5,6], Sergey V. Baykov [1,]* and Mikhail Krasavin [1,7,]*

1 Institute of Chemistry, Saint Petersburg State University, Saint Petersburg 199034, Russia
2 Pasteur Institute of Epidemiology and Microbiology, 14 Mira Street, Saint Petersburg 197101, Russia
3 Laboratory of Chemoinformatics and Bioinformatics, Sechenov First Moscow State Medical University, Moscow 119435, Russia
4 Saint Petersburg Research Institute of Phthisiopulmonology, 2-4 Ligovsky Prospekt, Saint Petersburg 191036, Russia
5 Peptide Chemistry Research Center, K. N. Toosi University of Technology, Tehran 19697, Iran
6 Medical Biology Research Center, Kermanshah University of Medical Sciences Kermanshah, Kermanshah 67155, Iran
7 School for Living Systems, Immanuel Kant Baltic Federal University, Kaliningrad 236041, Russia
* Correspondence: s.baykov@spbu.ru (S.V.B.); m.krasavin@spbu.ru (M.K.); Tel.: +7-931-3617-872 (M.K.); Fax: +7-812-428-6939 (M.K.)

Contents

Copies of ^{1}H and ^{13}C NMR spectra of compounds **4a-m** 2-15
Images of testing plates for lead compound **4a** 16

Copies of ^{1}H and ^{13}C NMR spectra.

^{1}H and ^{13}C NMR spectra of compound **4a**

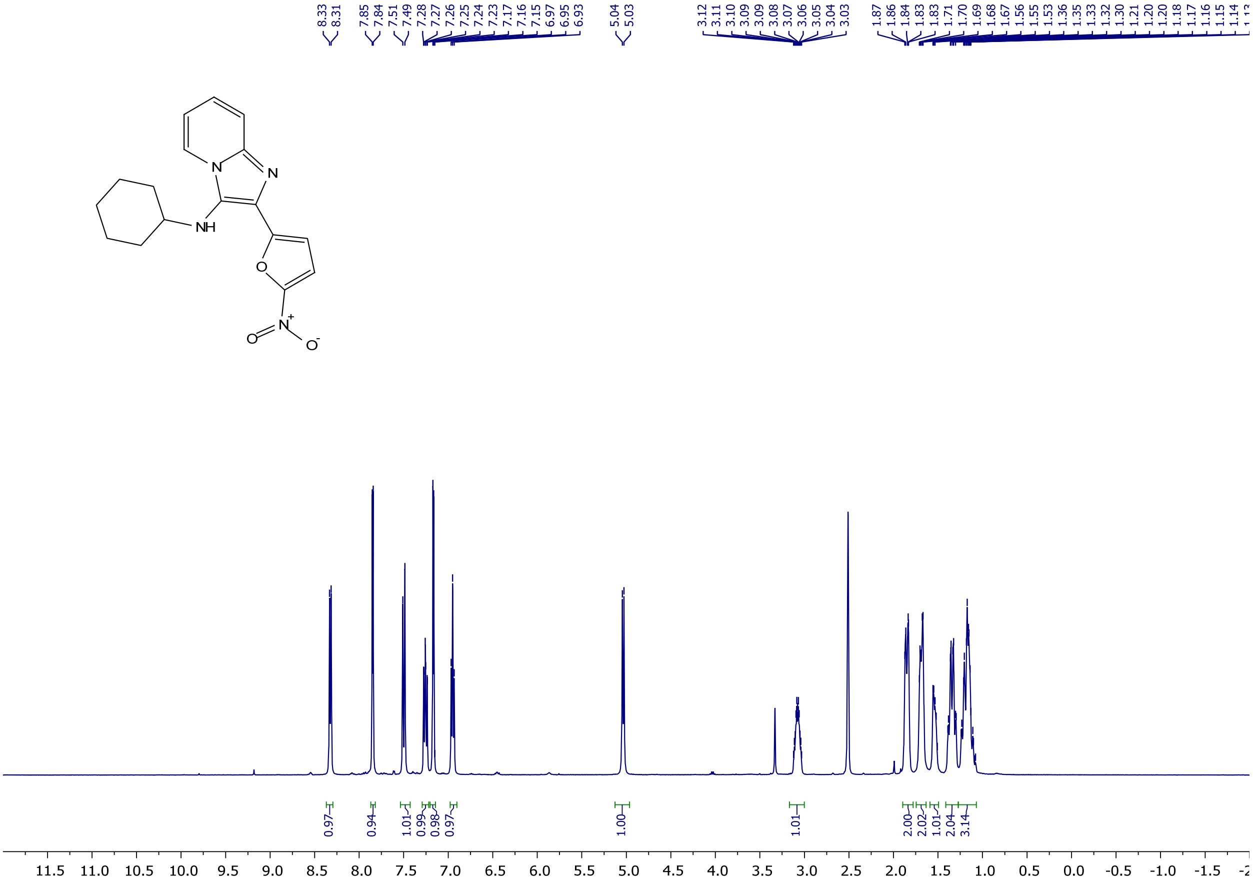

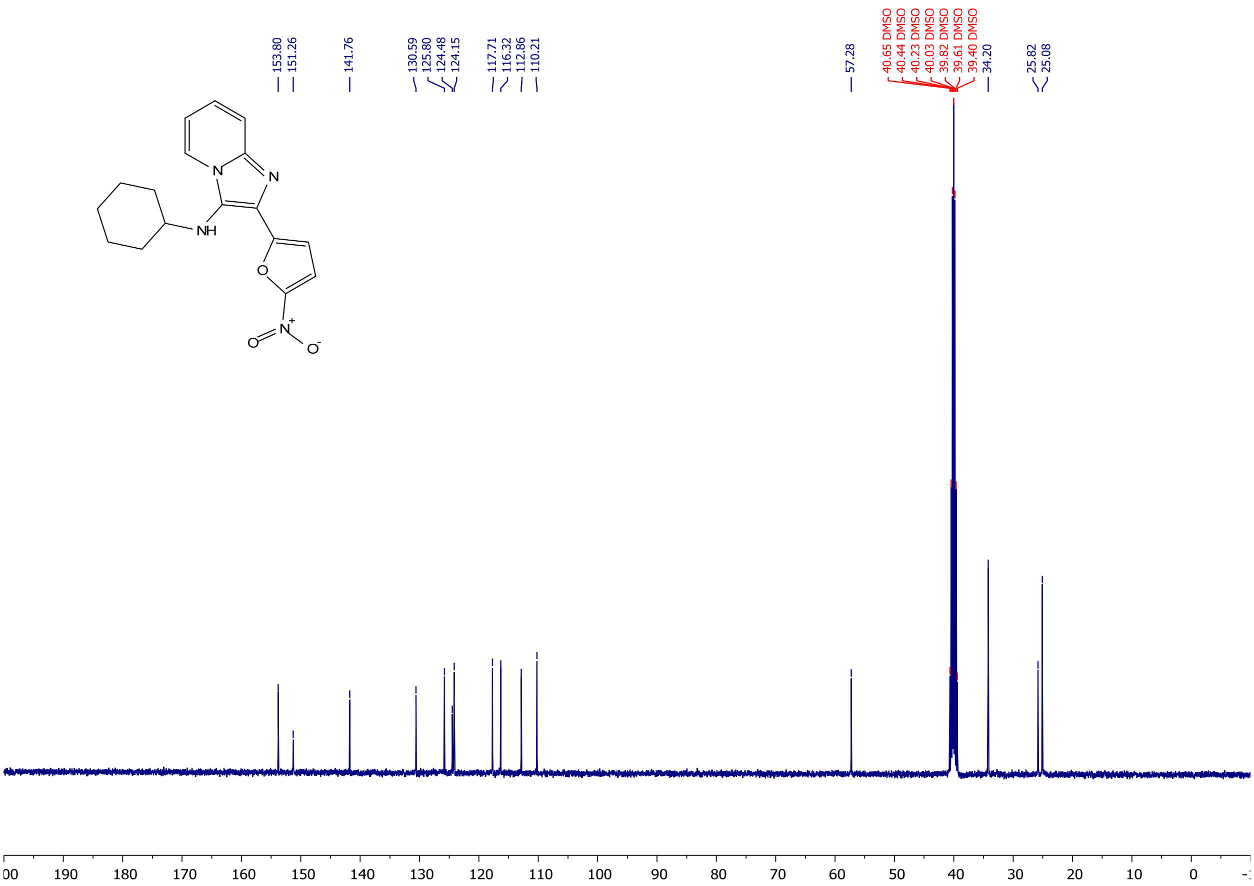

^{1}H and ^{13}C NMR spectra of compound **4b**

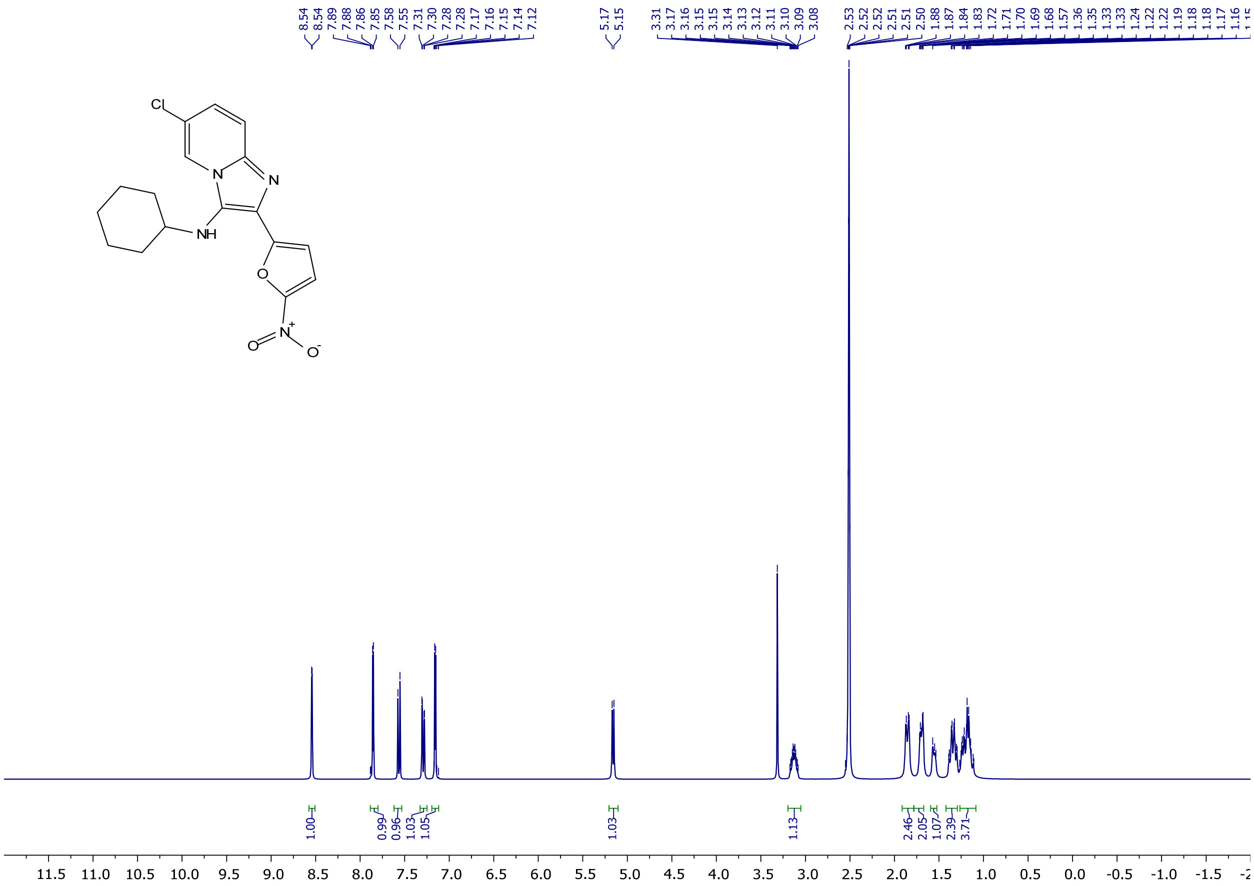

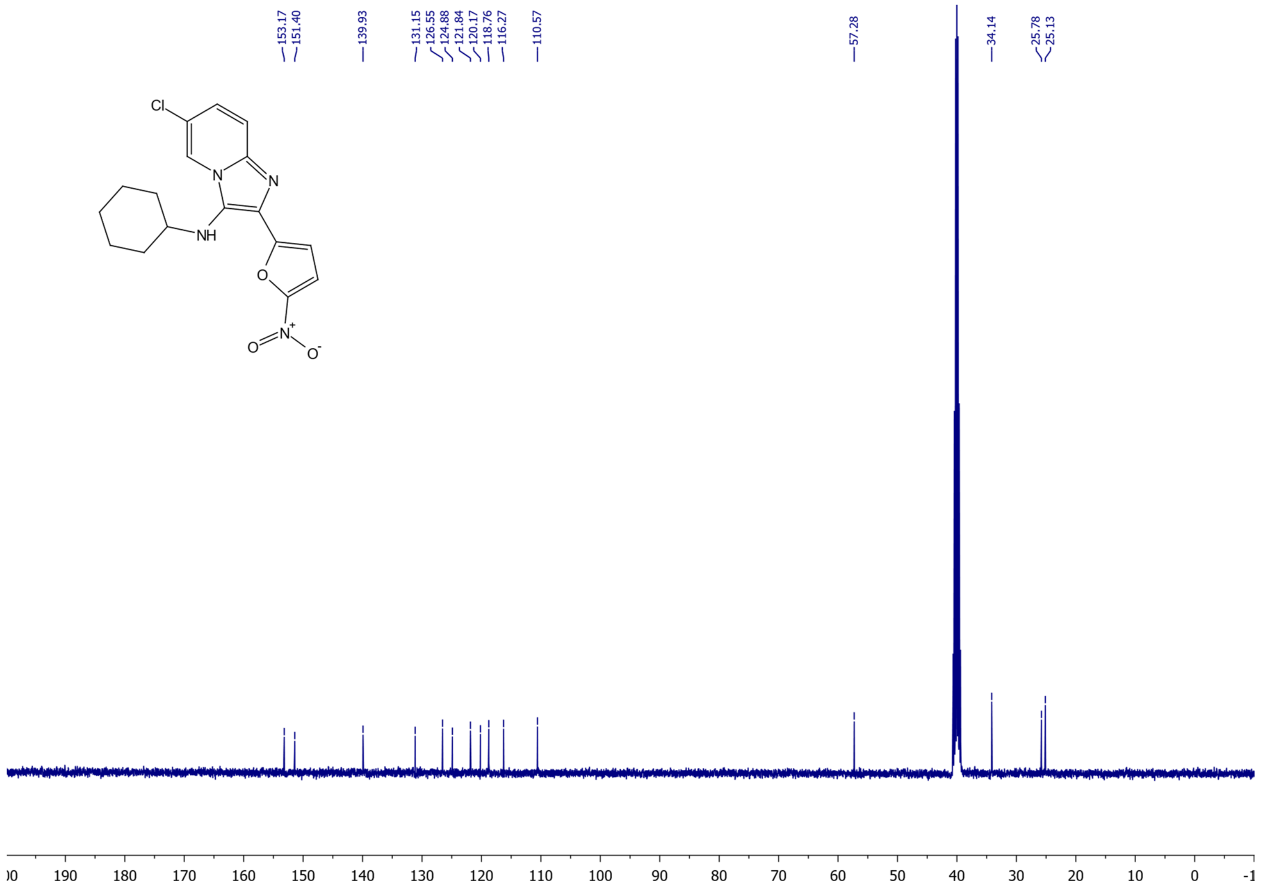

^1H and ^{13}C NMR spectra of compound **4c**

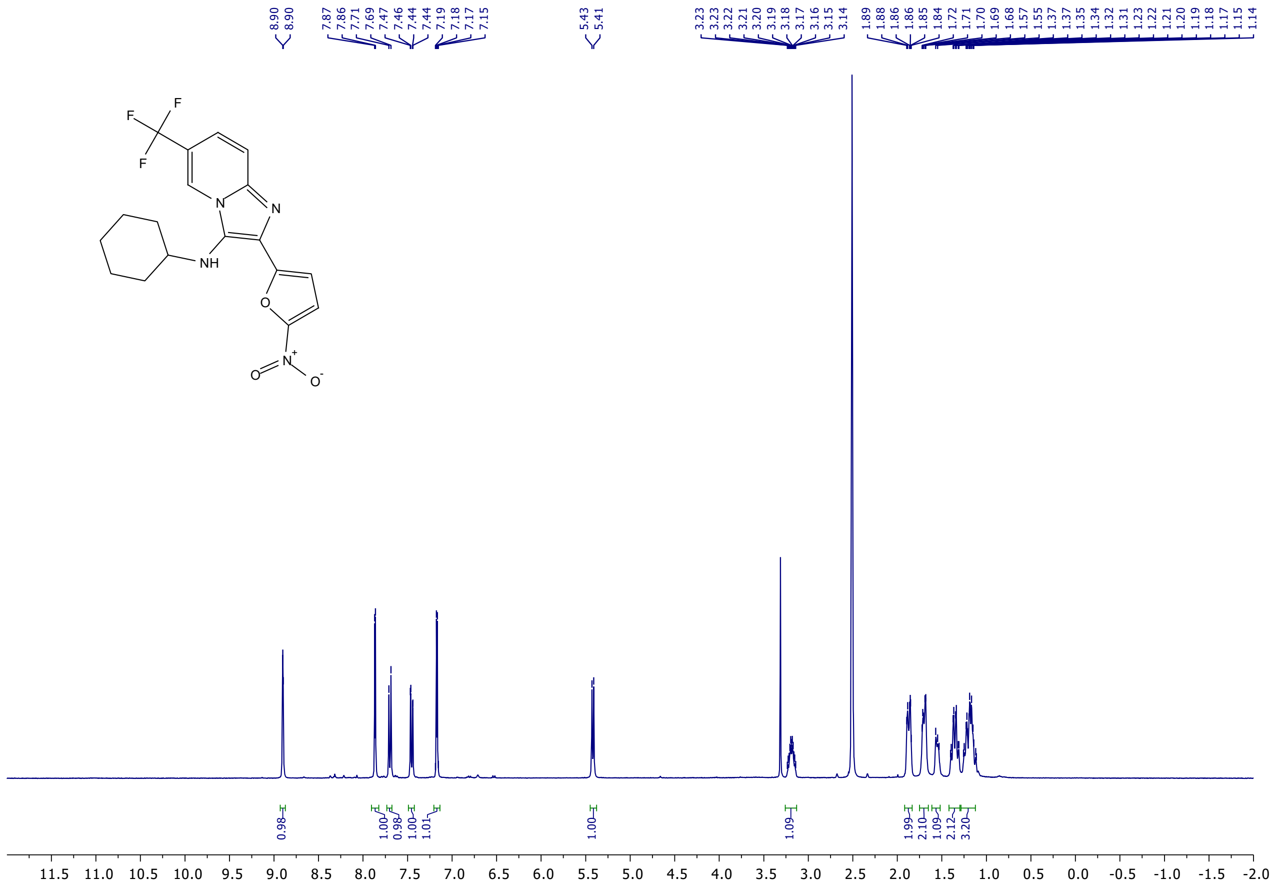

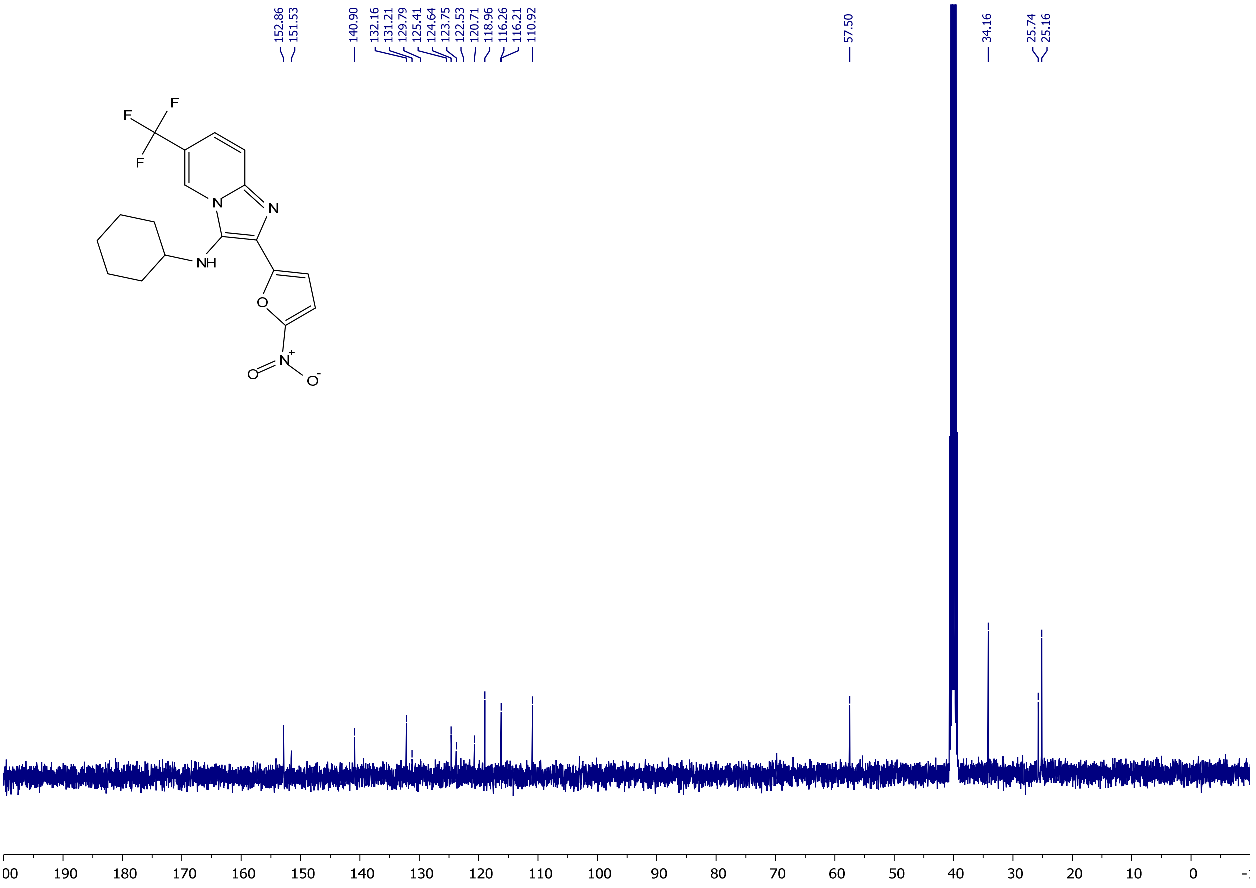
152.86
151.53
140.90
132.16
131.21
129.79
125.41
124.64
123.75
122.53
120.71
118.96
116.26
116.21
110.92
57.50
34.16
25.74
25.16
F
F
F
N
N
NH
O
N+
O
O-
190 180 170 160 150 140 130 120 110 100 90 80 70 60 50 40 30 20 10 0

^{1}H and ^{13}C NMR spectra of compound **4d**

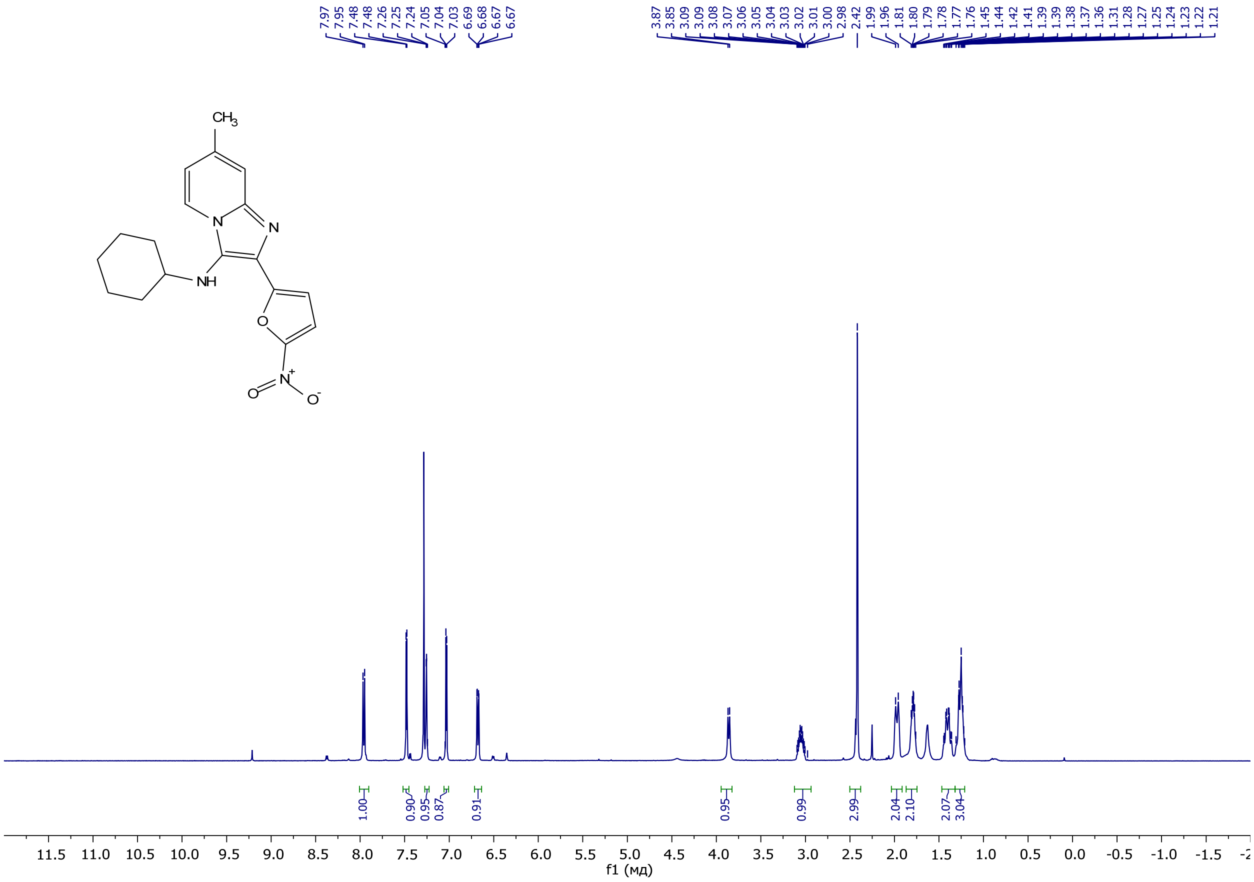

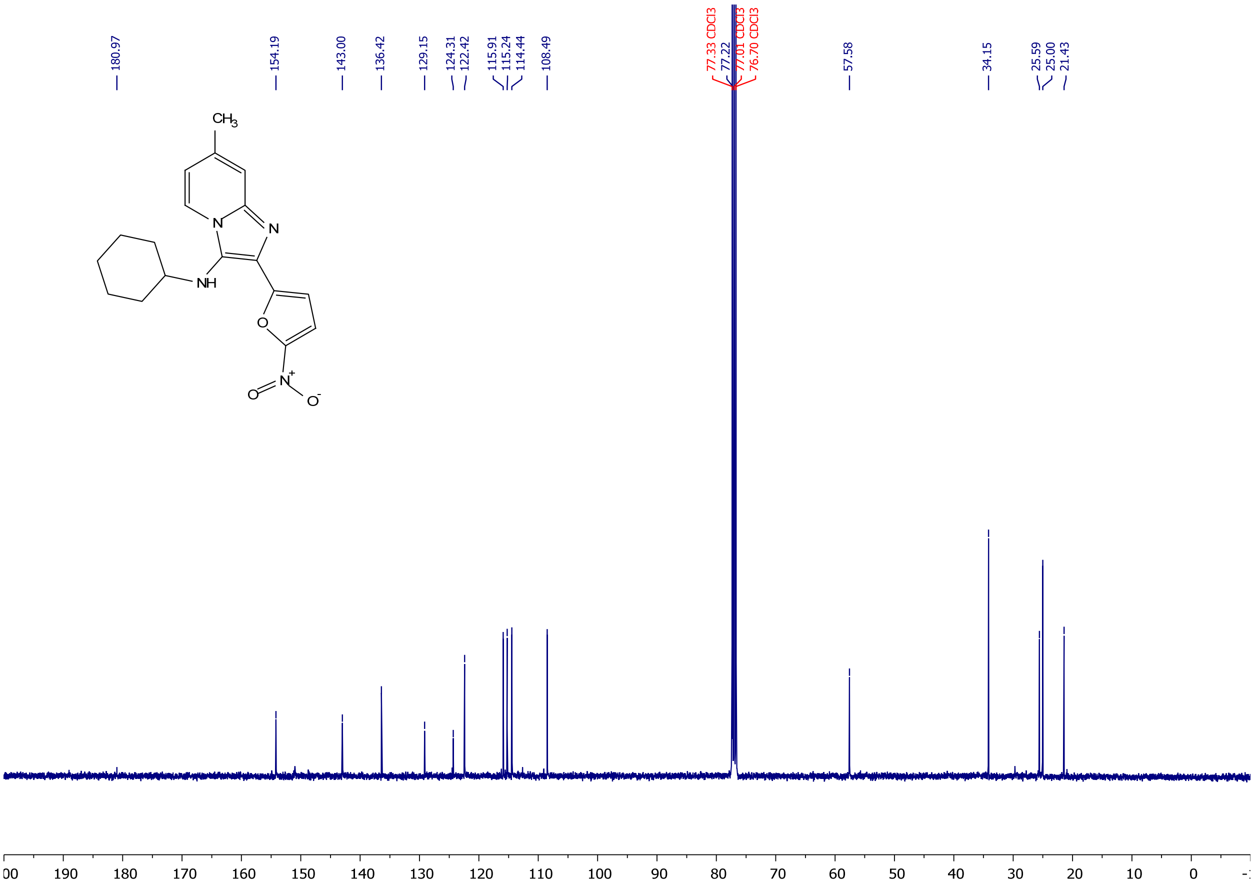

^{1}H and ^{13}C NMR spectra of compound **4e**

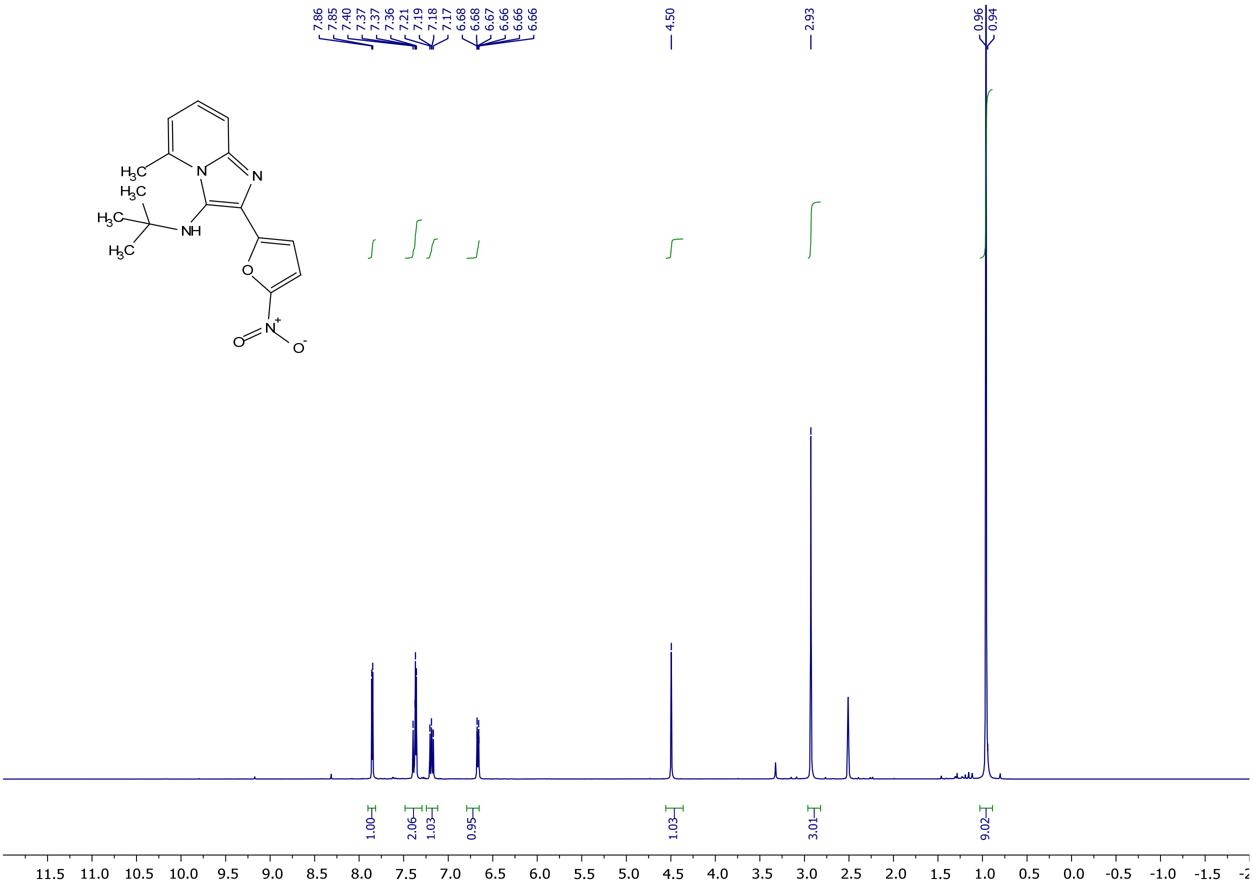

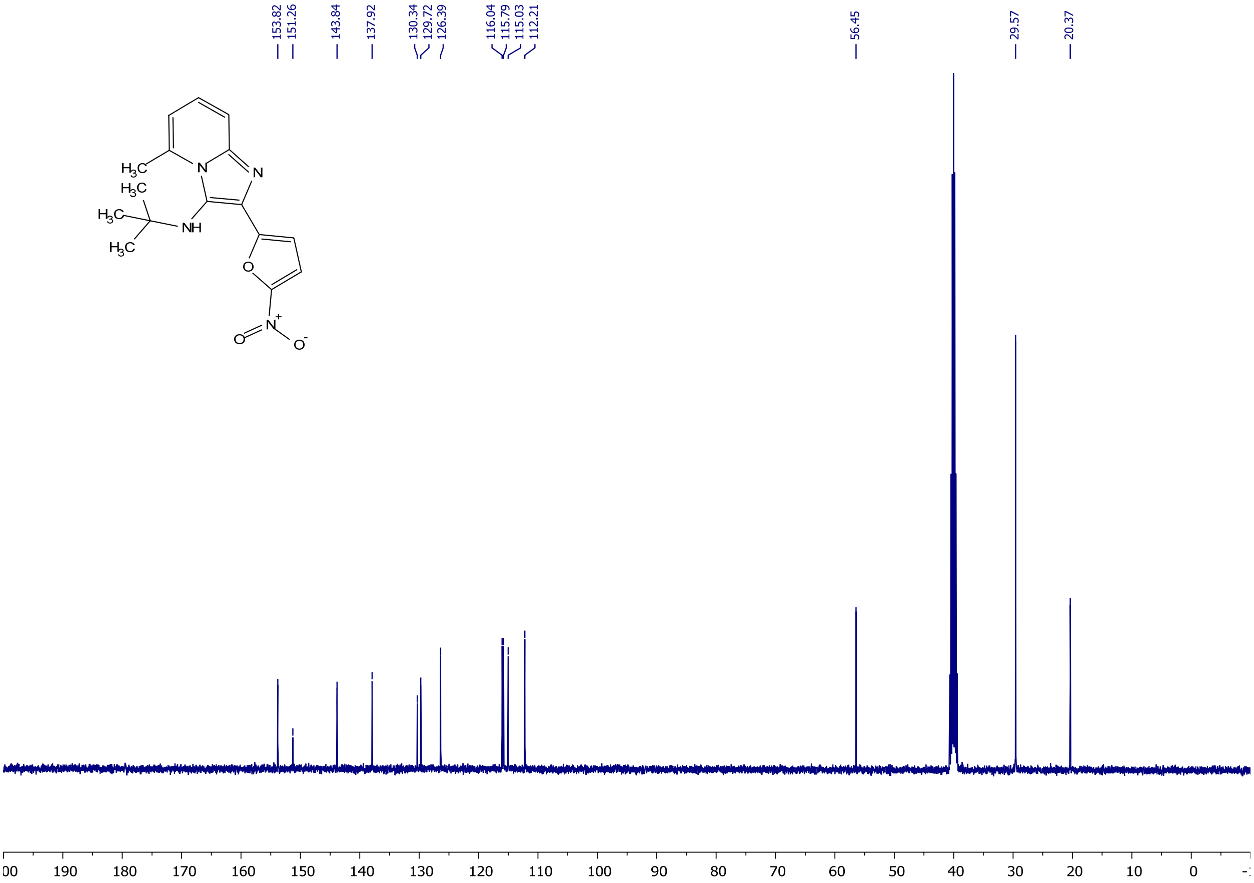

^{1}H and ^{13}C NMR spectra of compound **4f**

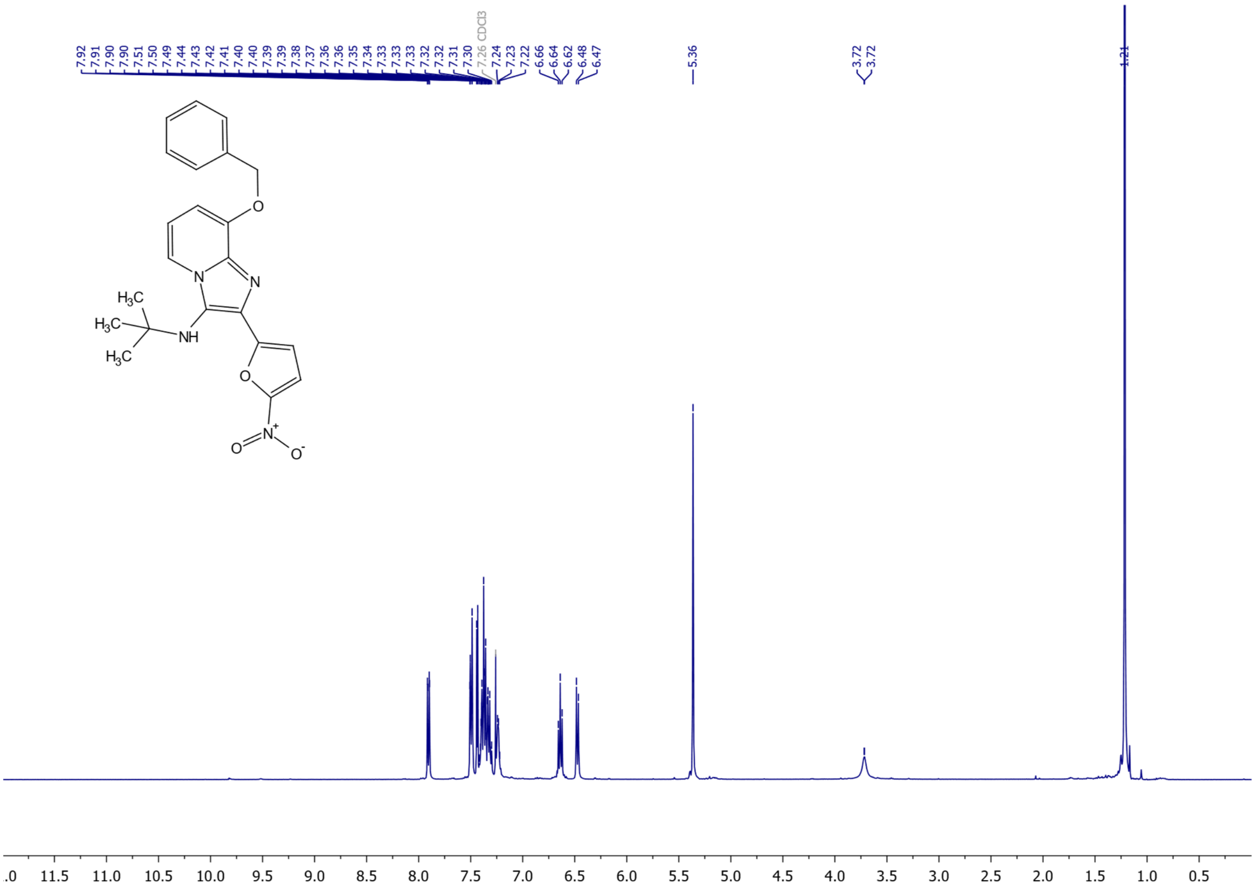

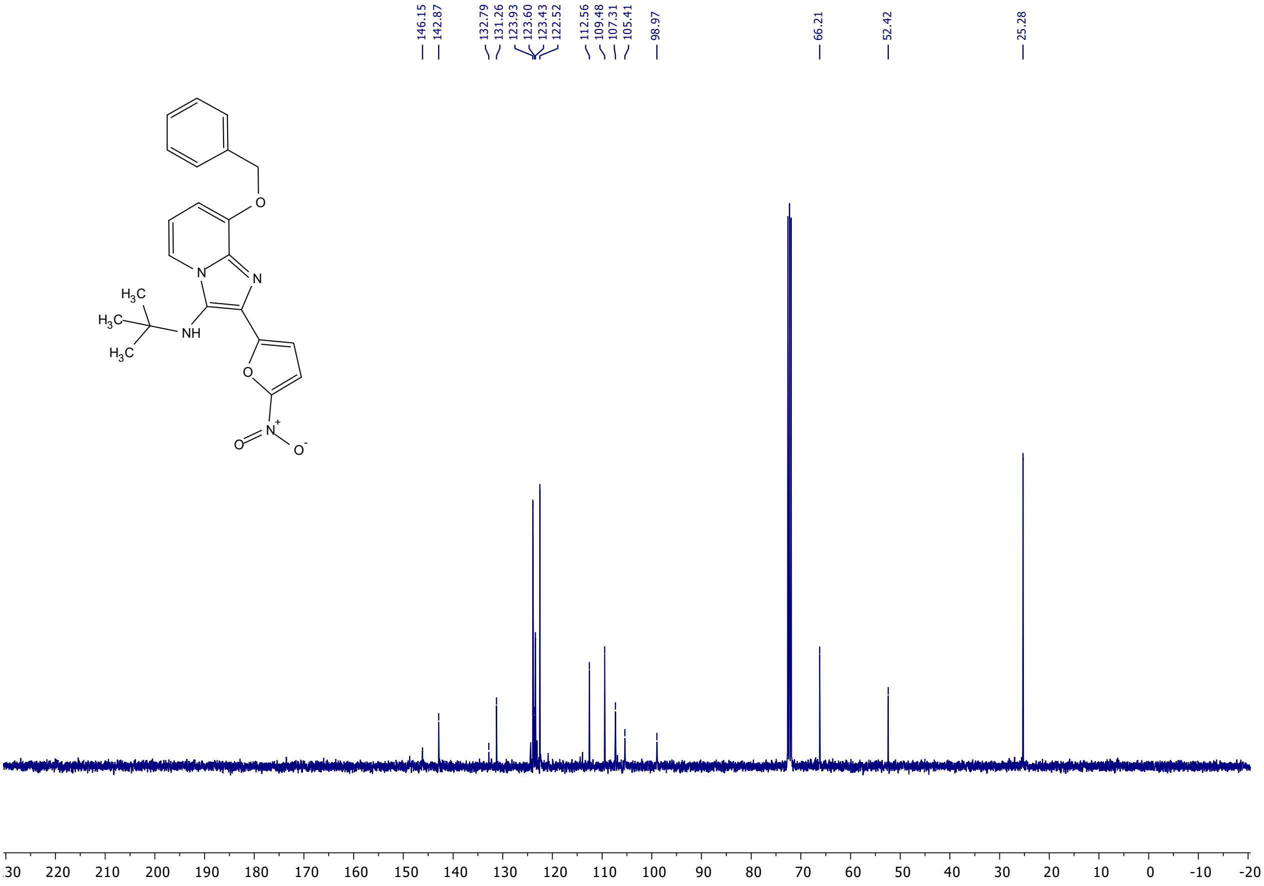

^{1}H and ^{13}C NMR spectra of compound **4g**

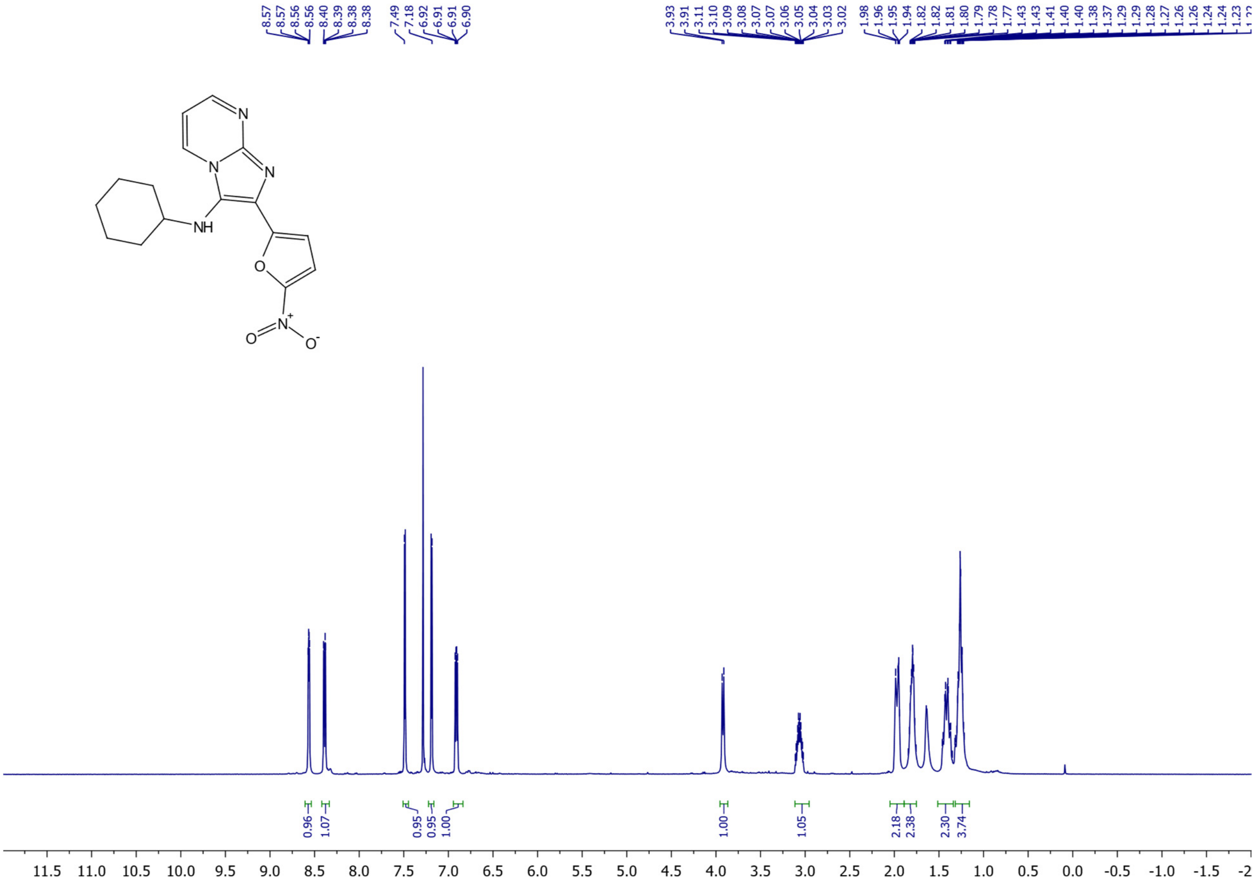

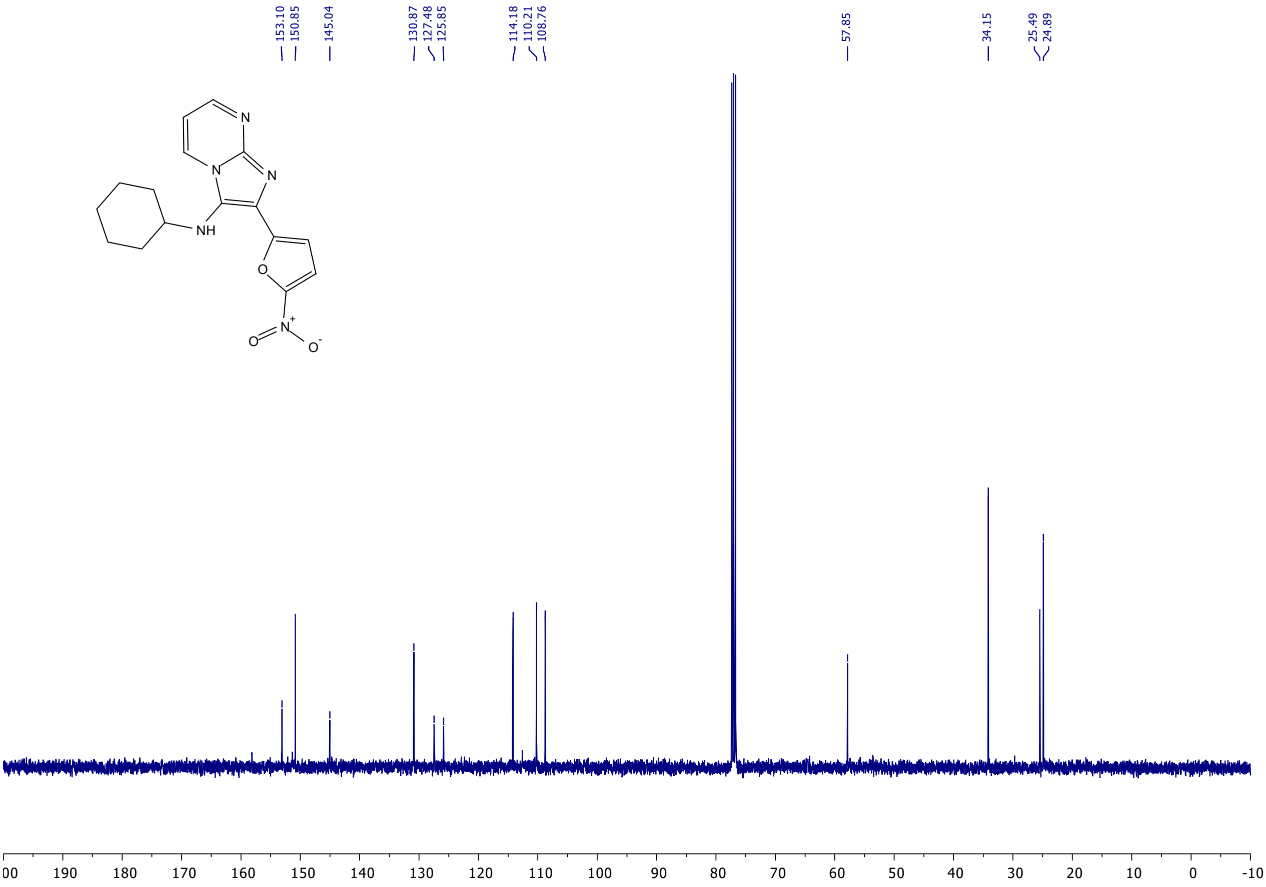

^{1}H and ^{13}C NMR spectra of compound **4h**

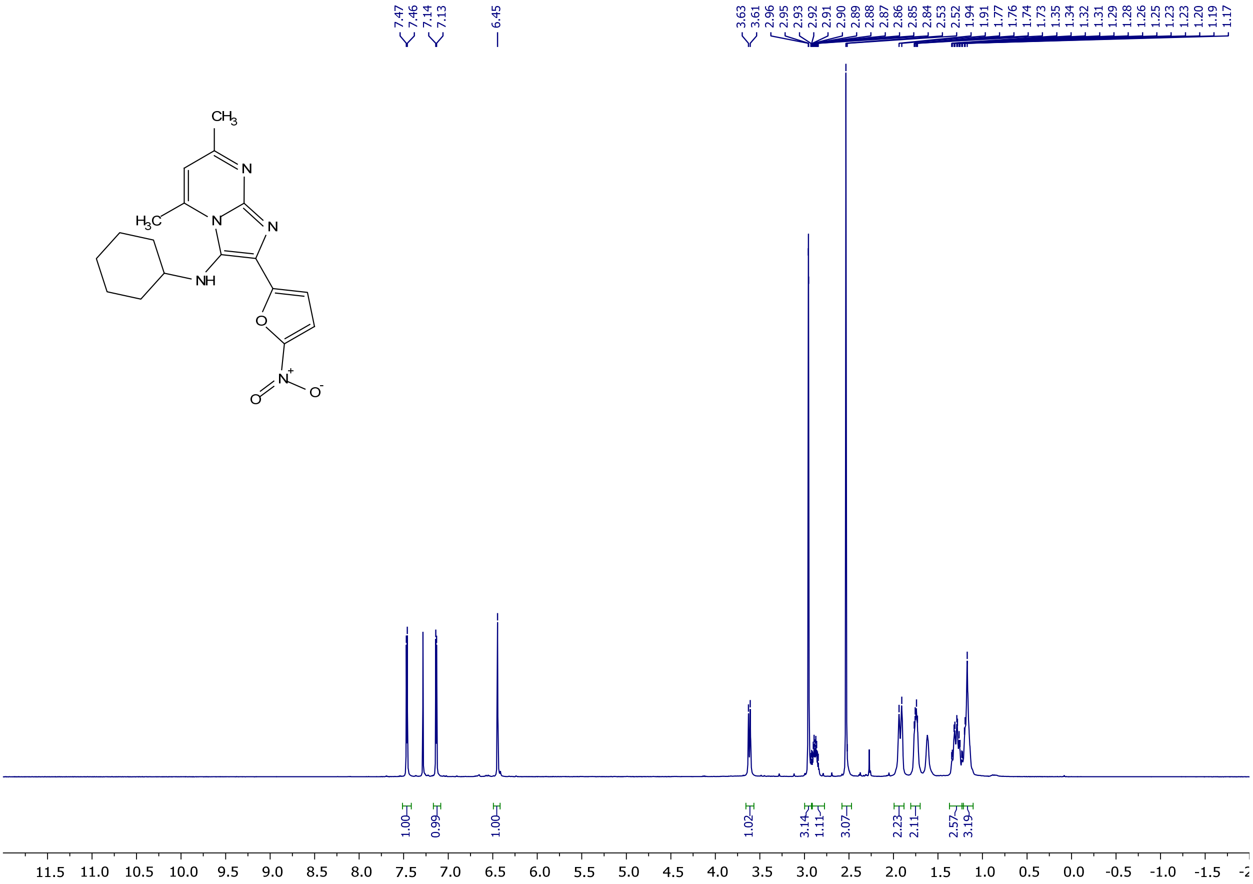

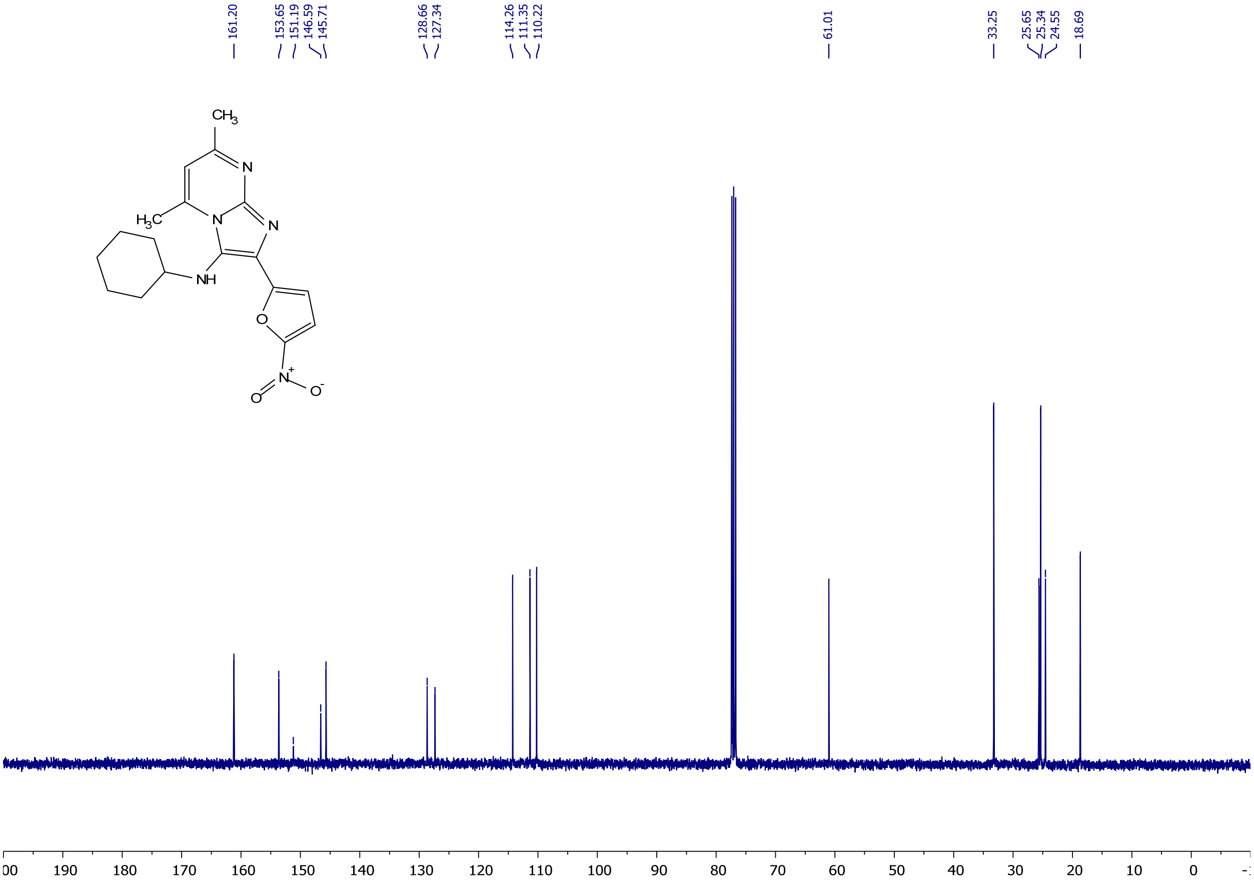

^{1}H and ^{13}C NMR spectra of compound **4i**

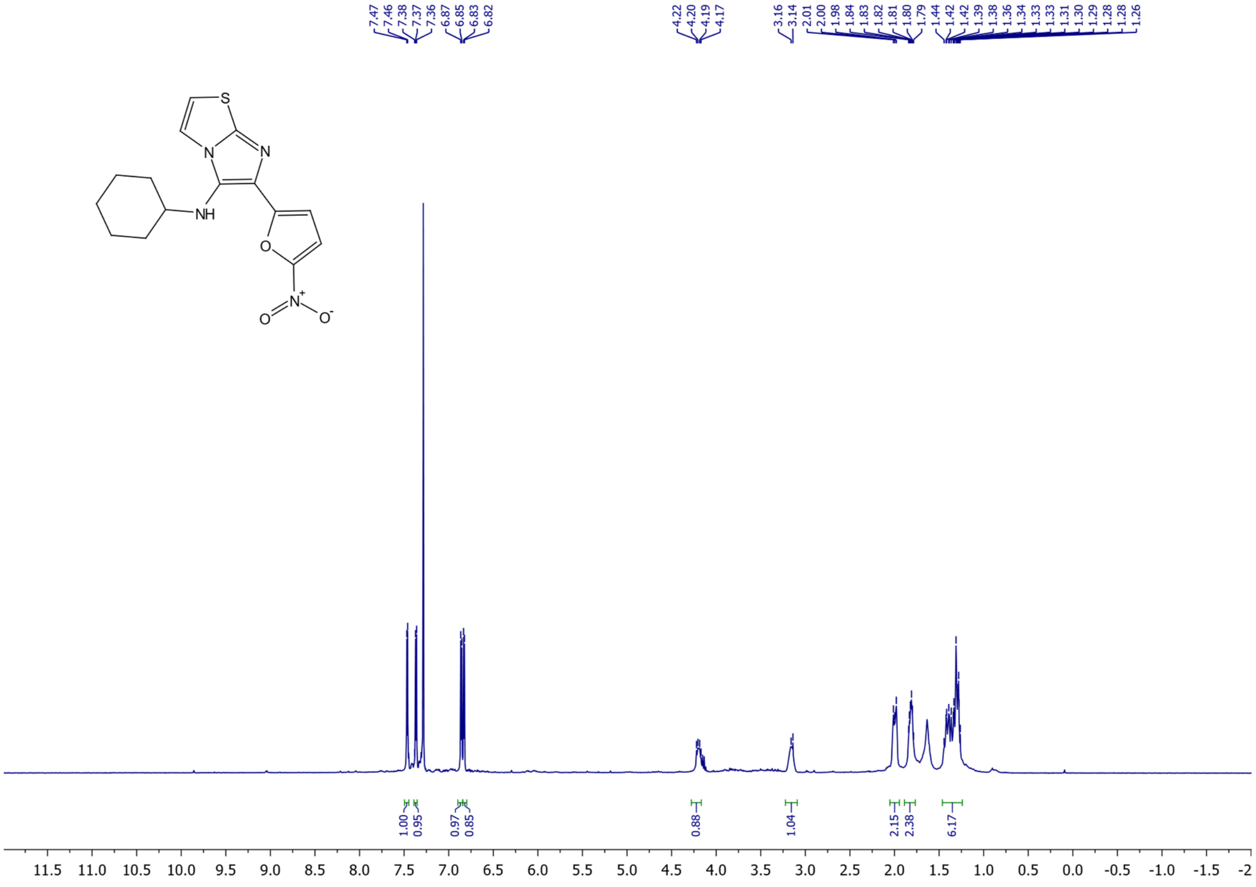

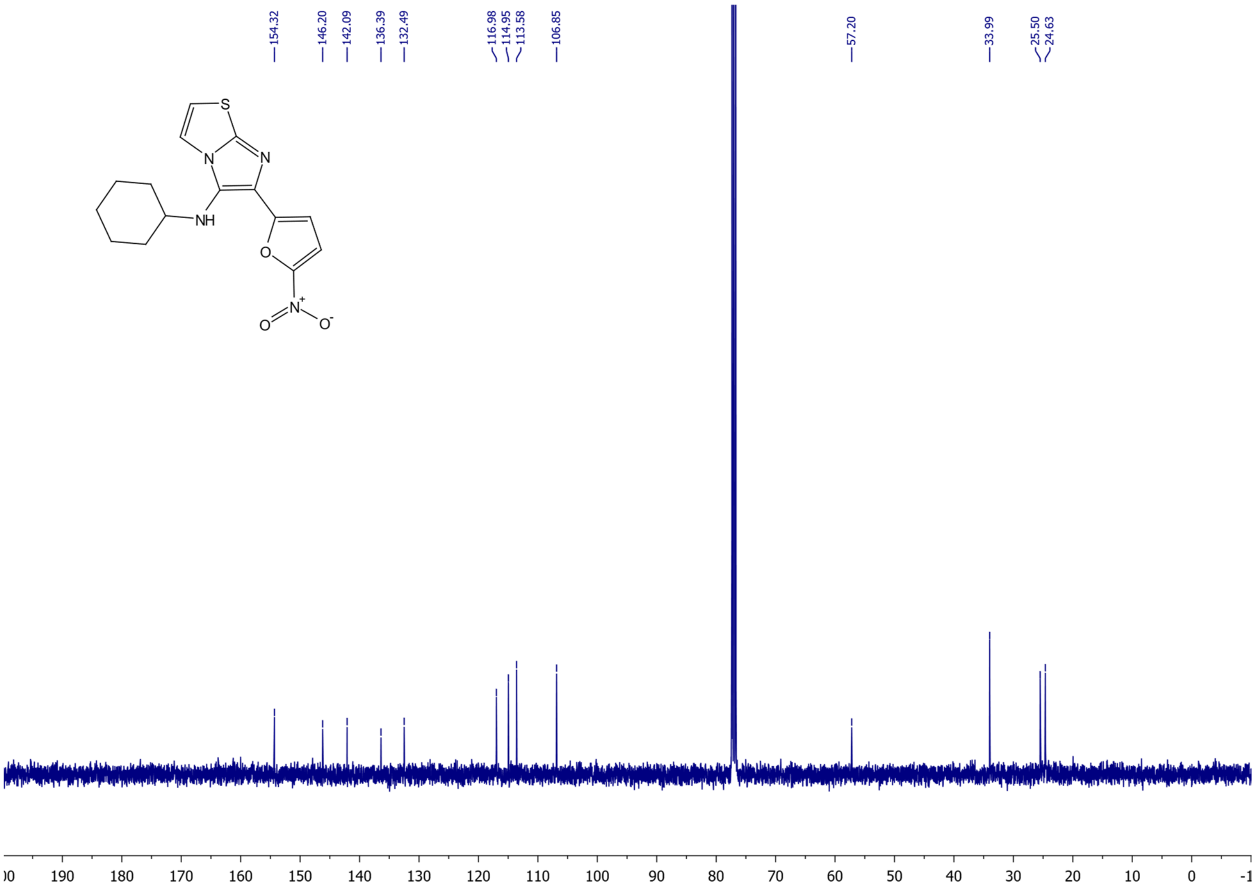

^{1}H and ^{13}C NMR spectra of compound **4j**

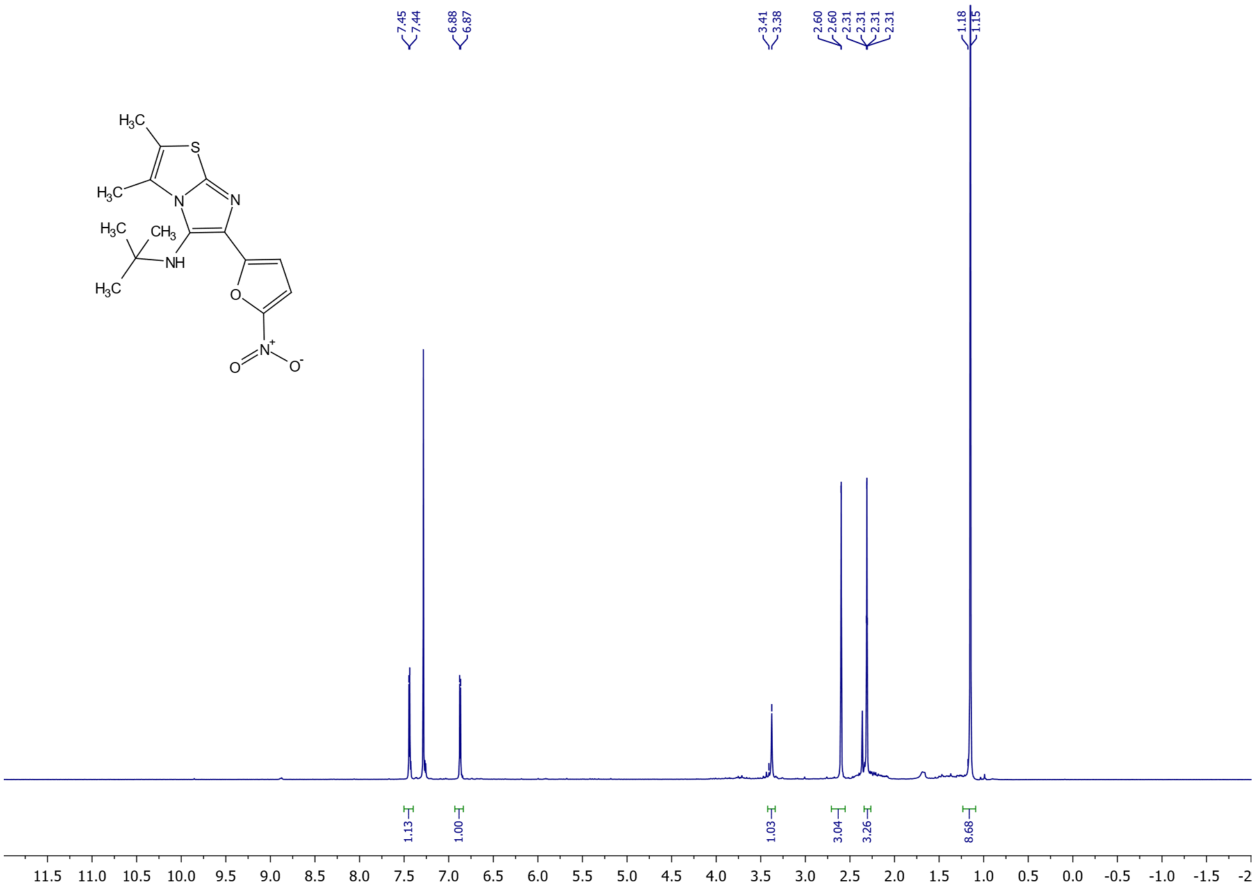

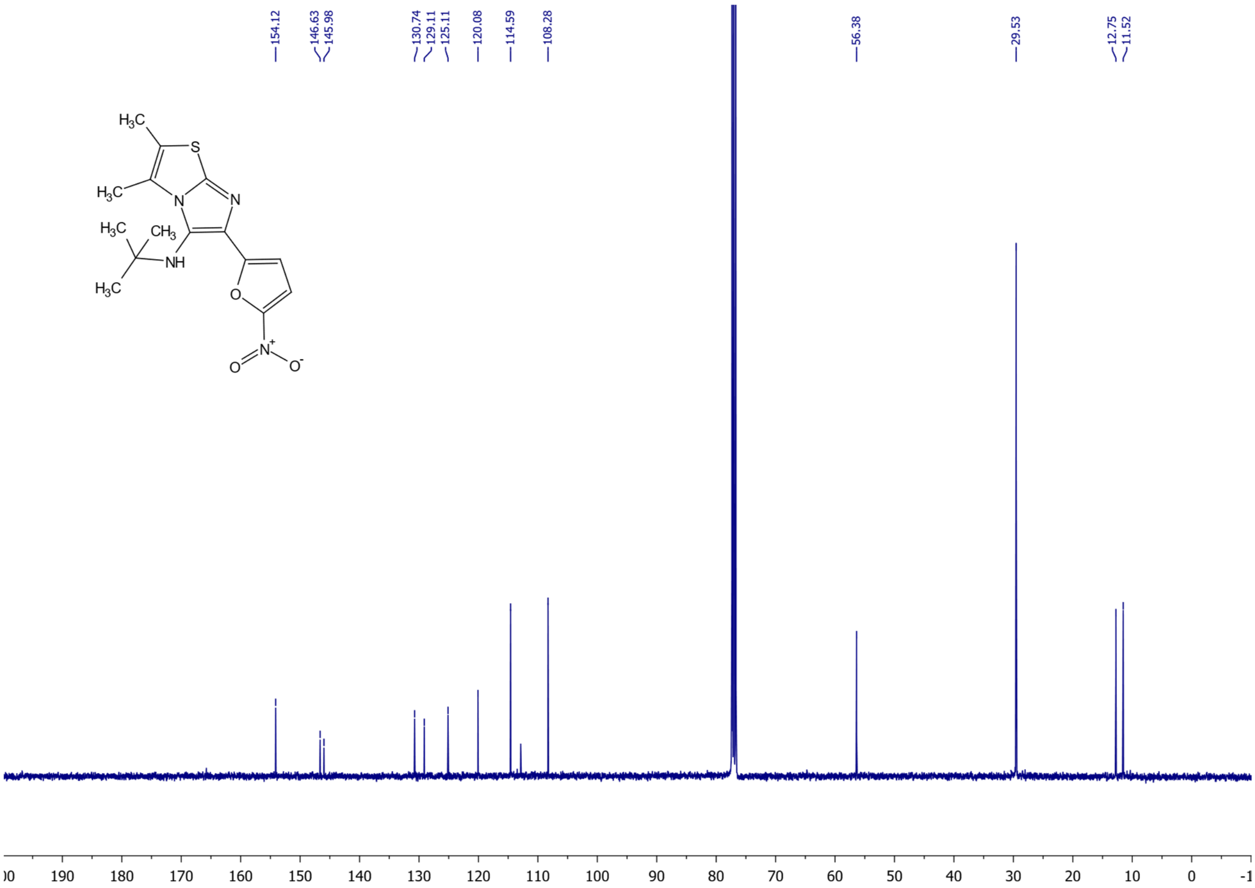

^{1}H and ^{13}C NMR spectra of compound **4k**

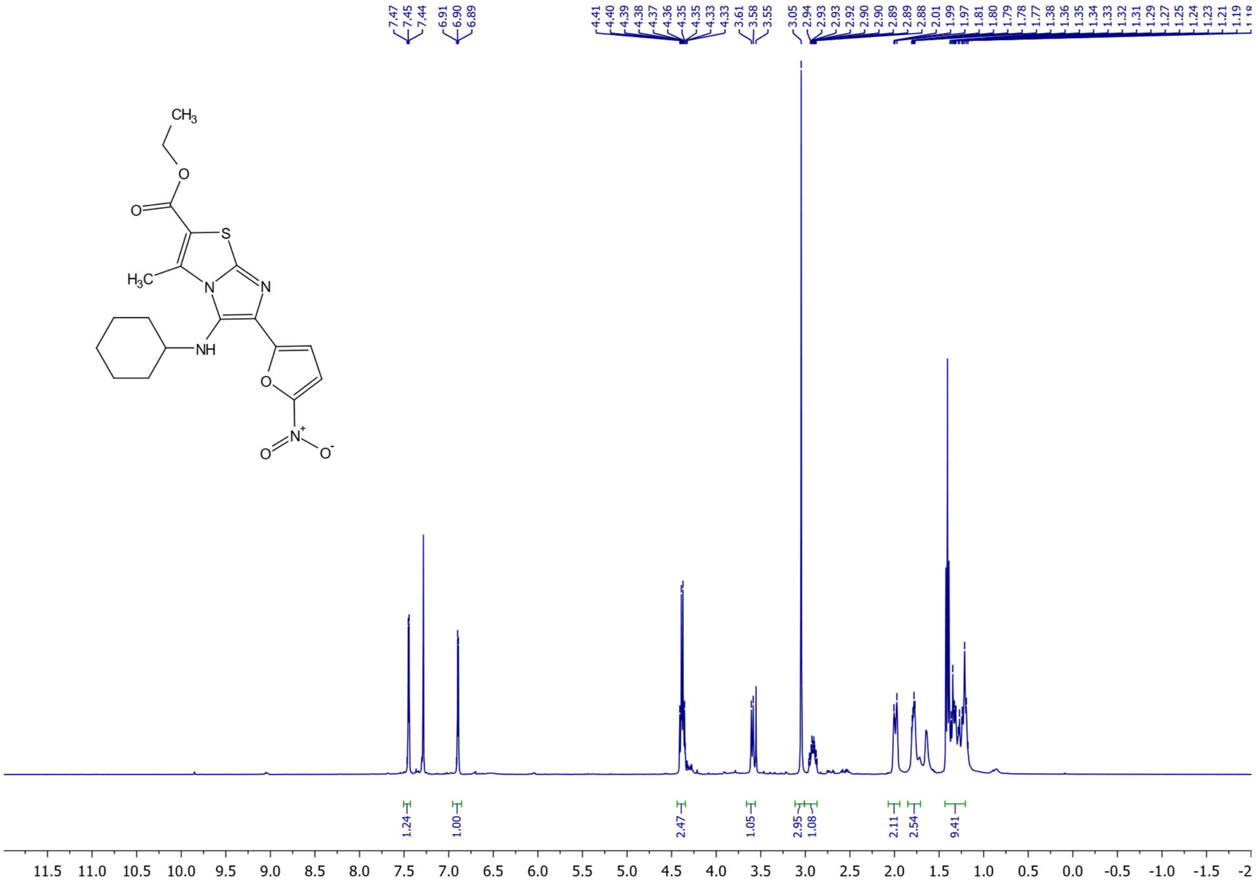

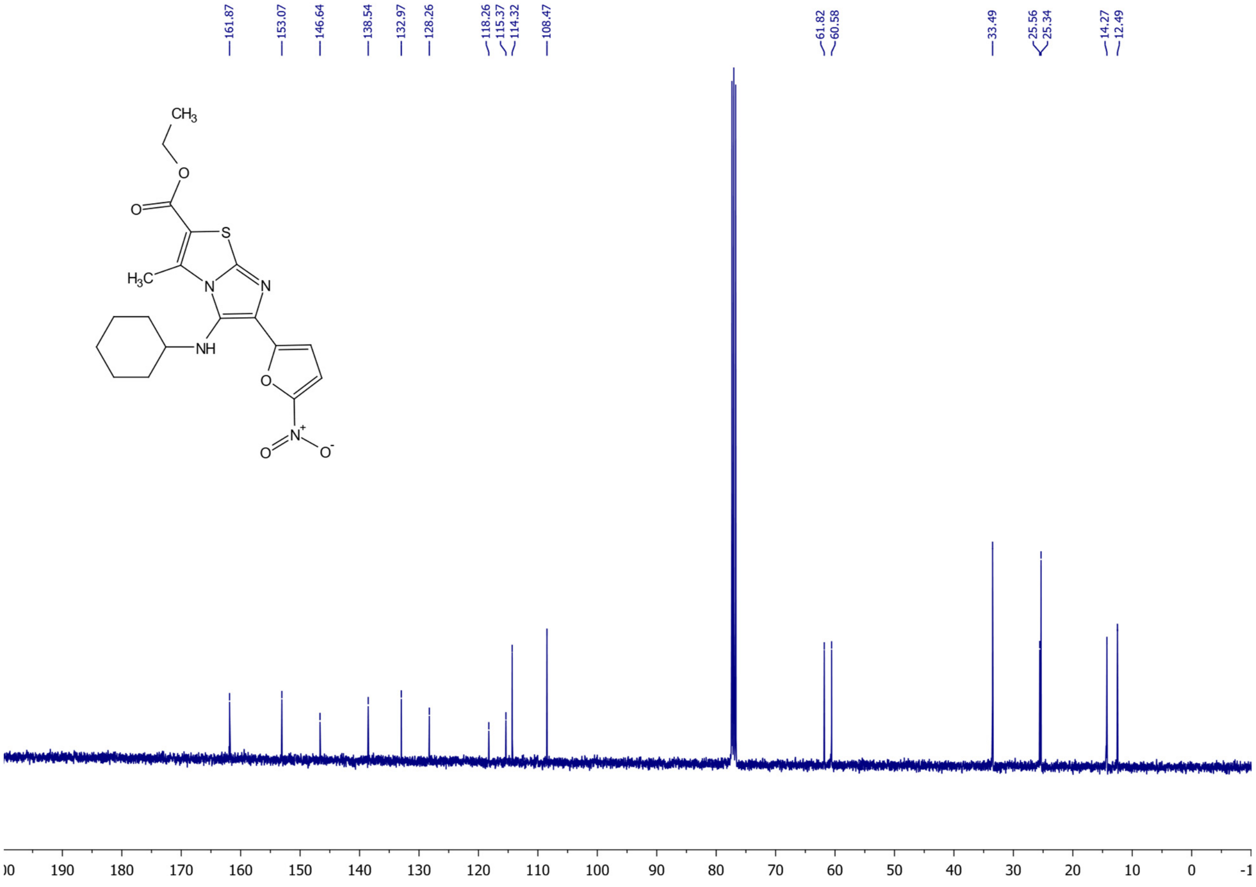

^{1}H and ^{13}C NMR spectra of compound **4l**

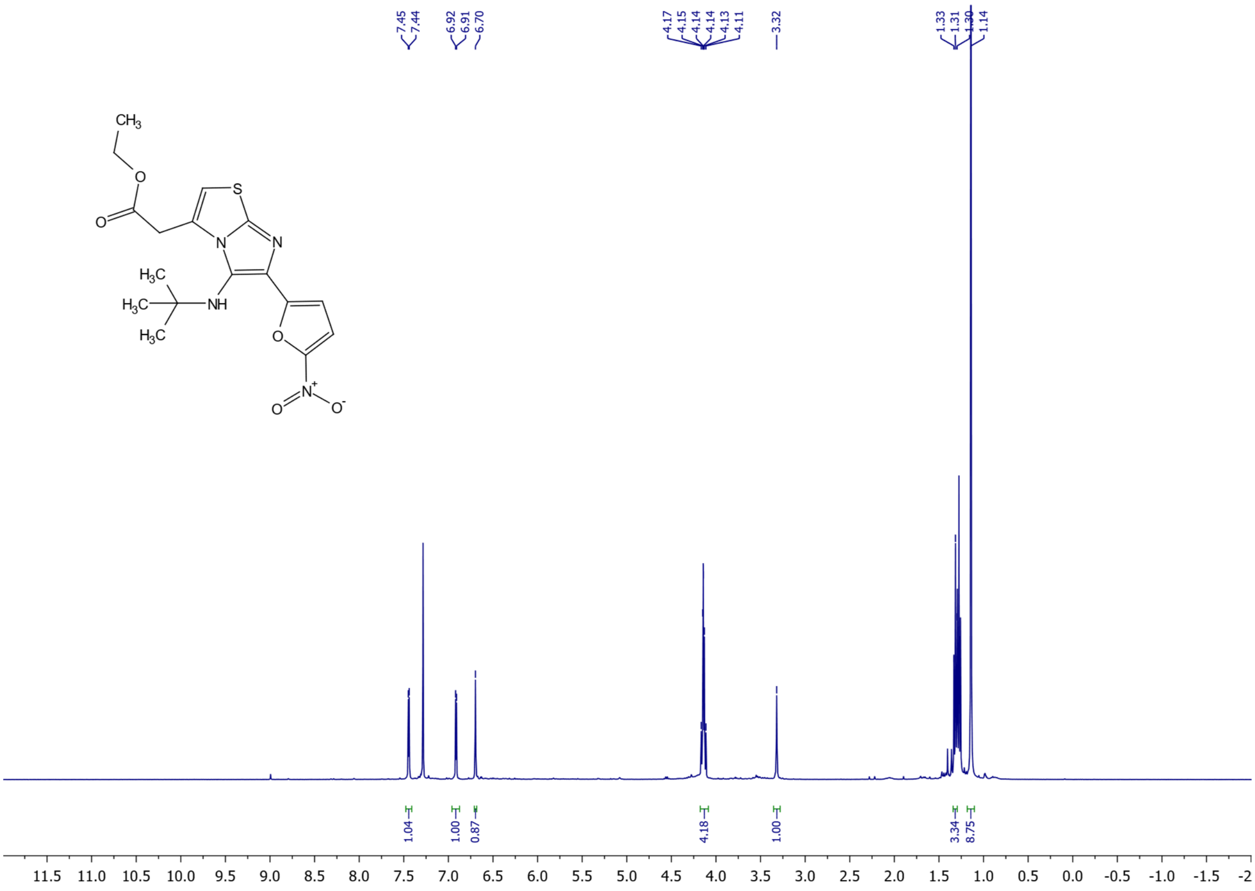

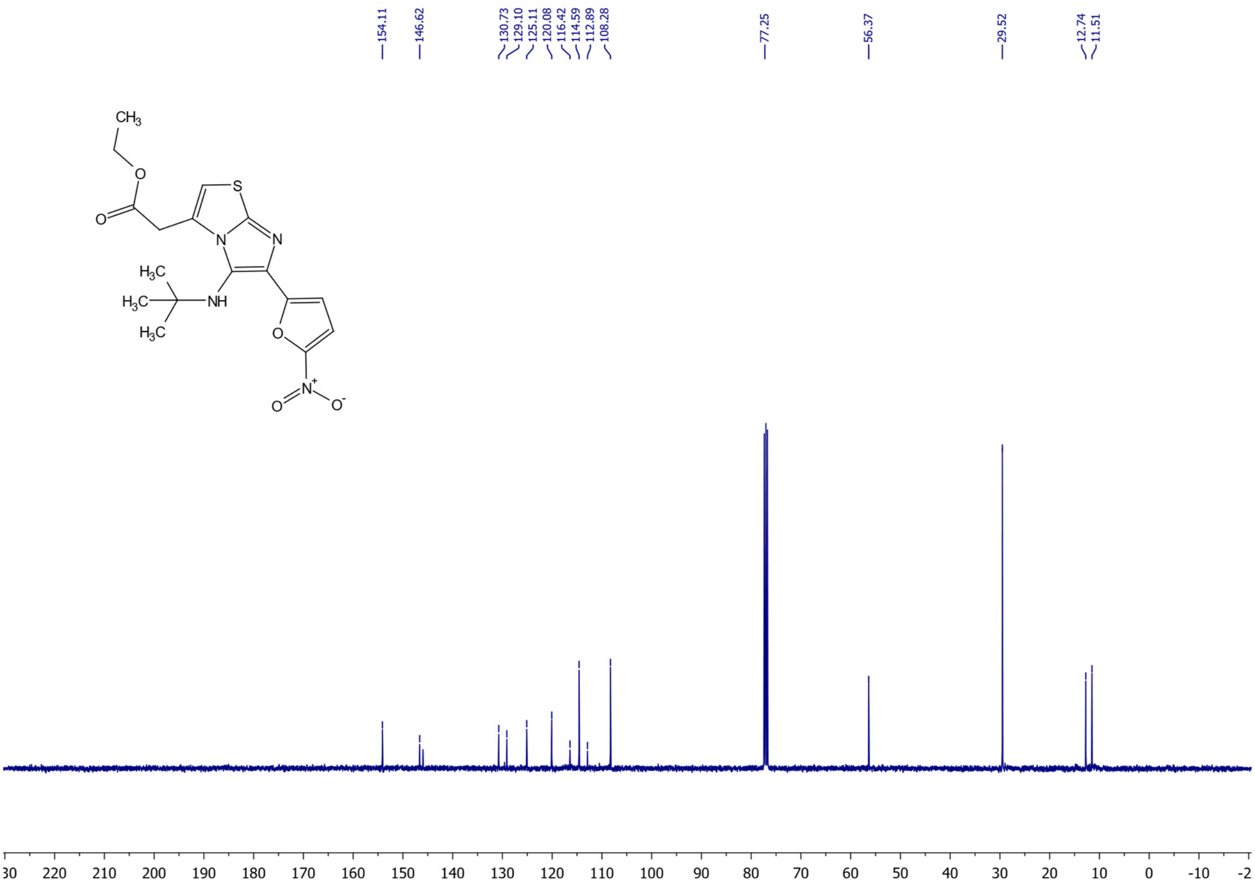

^{1}H and ^{13}C NMR spectra of compound **4m**

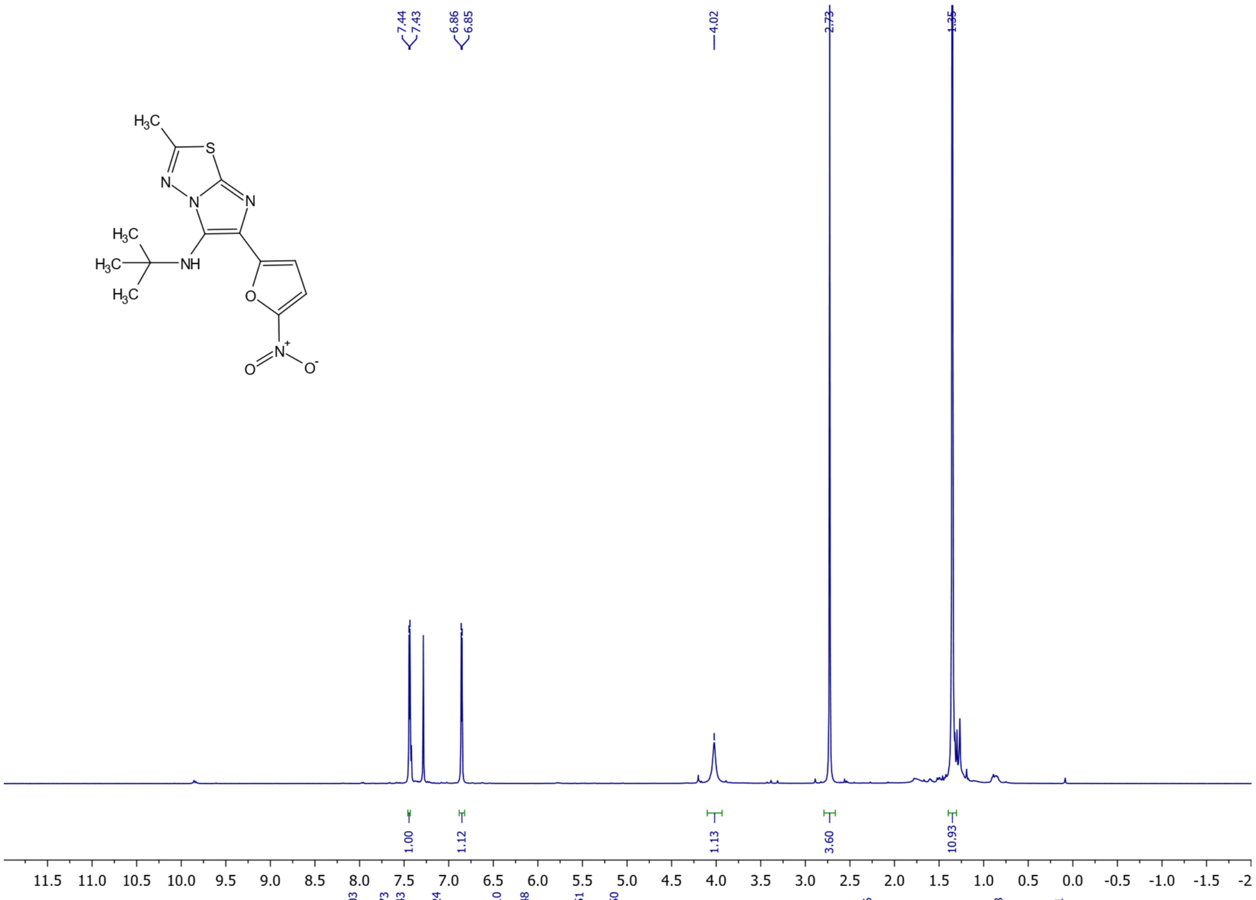

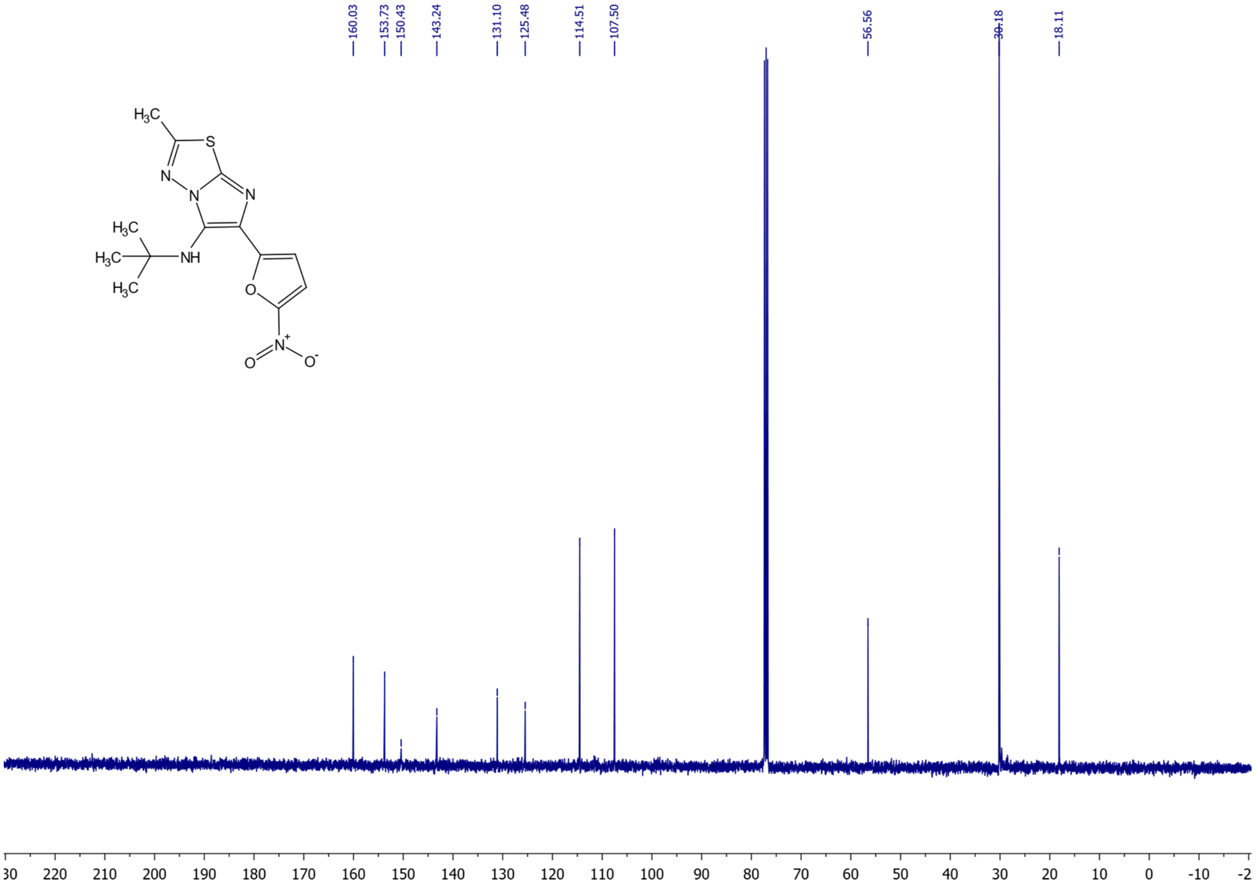

<u>Images of testing plates for lead compound **4a**</u>

Enterococcus faecalis ATCC 29812 (row 1, columns 1 through 6), 1 repeat out of 3
Staphylococcus aureus ATCC 25912 (row 2, columns 1 through 6), 1 repeat out of 3
Klebsiella pneumoniae ATCC 19882 (row 5, columns 1 through 6), 1 repeat out of 3
Enterobacter cloacae 13048 (row 6, columns 1 through 6), 1 repeat out of 3

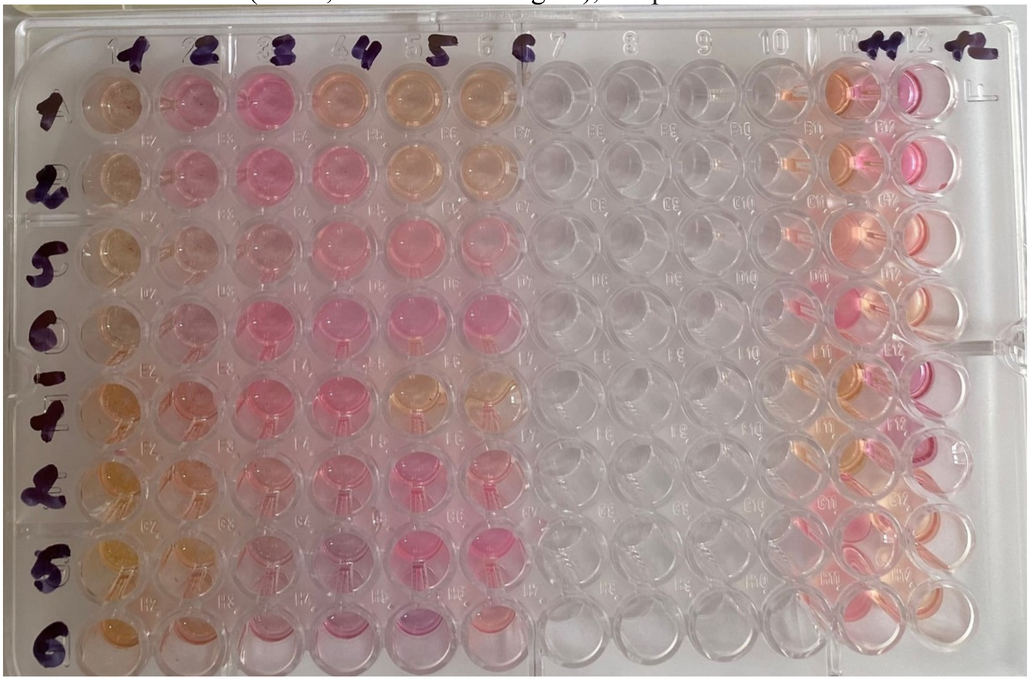

Mycobacterium tuberculosis H37Rv

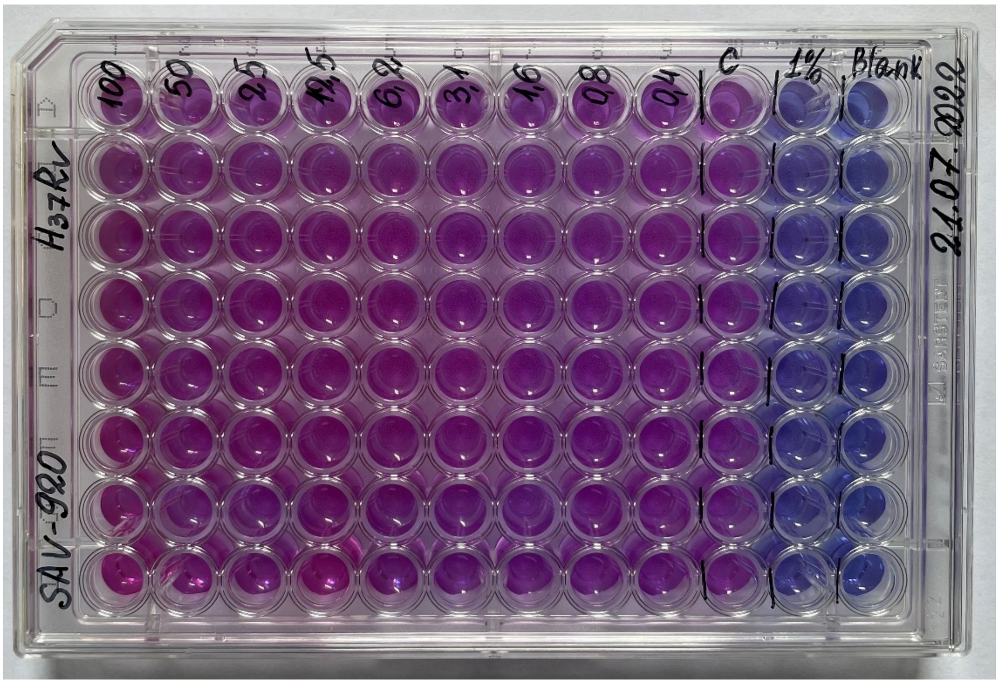

The only compound active against *M. tuberculosis* (**4i**, MIC 6.2 mg/mL):

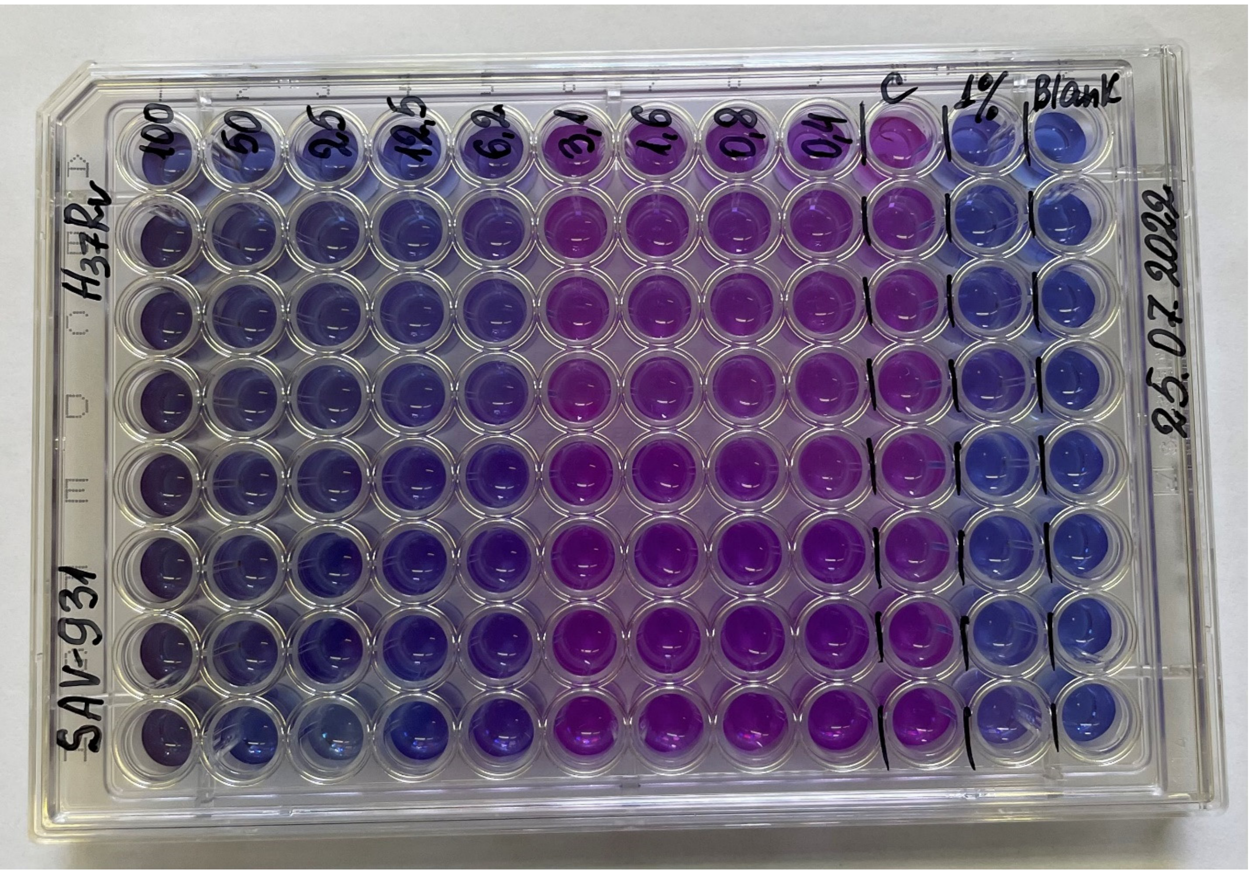

Supplement: Supplementary file 1 [file biomedicines-10-02203-s001.zip › biomedicines-1877157-supplementary.pdf]
